# Genome-wide analysis of expansin superfamily in wild *Arachis* discloses a stress-responsive expansin-like B gene

**DOI:** 10.1007/s11103-017-0594-8

**Published:** 2017-02-27

**Authors:** Larissa Arrais Guimaraes, Ana Paula Zotta Mota, Ana Claudia Guerra Araujo, Lucio Flavio de Alencar Figueiredo, Bruna Medeiros Pereira, Mario Alfredo de Passos Saraiva, Raquel Bispo Silva, Etienne G. J. Danchin, Patricia Messenberg Guimaraes, Ana Cristina Miranda Brasileiro

**Affiliations:** 1 Embrapa Recursos Genéticos e Biotecnologia, Parque Estação Biológica, Final W5 Norte, Brasília, DF CP 02372 Brazil; 2 Universidade do Rio Grande do Sul, Porto Alegre, RS Brazil; 3 Universidade de Brasília, Campus Darcy Ribeiro, Brasília, DF Brazil; 4 Institut Sophia Agrobiotech, INRA, University of Nice Sophia Antipolis, CNRS, 06900 Sophia Antipolis, France

**Keywords:** Drought, *Meloidogyne*, Ultraviolet (UV), Composite plant, *Glycine max*

## Abstract

Expansins are plant cell wall-loosening proteins involved in adaptive responses to environmental stimuli and various developmental processes. The first genome-wide analysis of the expansin superfamily in the *Arachis* genus identified 40 members in *A. duranensis* and 44 in *A. ipaënsis*, the wild progenitors of cultivated peanut (*A. hypogaea*). These expansins were further characterized regarding their subfamily classification, distribution along the genomes, duplication events, molecular structure, and phylogeny. A RNA-seq expression analysis in different *Arachis* species showed that the majority of these expansins are modulated in response to diverse stresses such as water deficit, root-knot nematode (RKN) infection, and UV exposure, with an expansin-like B gene (*AraEXLB8*) displaying a highly distinct stress-responsive expression profile. Further analysis of the *AraEXLB8* coding sequences showed high conservation across the *Arachis* genotypes, with eight haplotypes identified. The modulation of *AraEXLB8* expression in response to the aforementioned stresses was confirmed by qRT-PCR analysis in distinct *Arachis* genotypes, whilst in situ hybridization revealed transcripts in different root tissues according to the stress imposed. The overexpression of *AraEXLB8* in soybean (*Glycine max*) composite plants remarkably decreased the number of galls in transformed hairy roots inoculated with RKN. This study improves the current understanding of the molecular evolution, divergence, and gene expression of expansins in *Arachis*, and provides molecular and functional insights into the role of expansin-like B, the less-studied plant expansin subfamily.

**Electronic supplementary material:**

The online version of this article (doi:10.1007/s11103-017-0594-8) contains supplementary material, which is available to authorized users.

## Introduction

Expansins are cell wall loosening proteins involved in extension and relaxation of cells in growing tissues through a non-enzymatic activity (McQueen-Mason et al. 1992). Plant expansins are also implicated in responses to many abiotic and biotic stresses, such as drought; salinity; cold; heat; oxidative stress; herbivore attack and phytopathogen infection (Marowa et al. 2016), indicating that these proteins constitute a common component in the response of plants to stress. The role of expansins in stress responses has been reinforced by studies showing that the ectopic overexpression in transgenic plants of some expansin-coding genes leads, in general, to enhanced overall plant growth and tolerance to abiotic stress (Cosgrove 2015; Marowa et al. 2016; Sasidharan et al. 2011). In contrast, the reduction of expansin transcripts, which inhibits cell expansion, leads to an increase in resistance to plant diseases possibly due to a more efficient cell wall physical protection (Ding et al. 2008; Gal et al. 2006). Expansins are also involved in responses to oxidative stress (Han et al. 2015) derived from enhanced production and accumulation of Reactive Oxygen Species (ROS) (Baxter et al. 2014). As a secondary stress, oxidative damage is generally ubiquitous in almost all types of stresses, including ultraviolet (UV) exposure, thus supporting the idea that expansins are common and crucial players in the response to multiple and simultaneous stresses. Nevertheless, the non-enzymatic mechanisms by which expansins influence the ability of plants to withstand associated stresses remain uncertain.

The expansin superfamily is ubiquitous in the plant kingdom and classified into four subfamilies: α-expansin (EXPA), β-expansin (EXPB), expansin-like A (EXLA) and expansin-like B (EXLB) (Kende et al. 2004). It has been assumed that expansin genes belonging to the same phylogenetic group share almost similar functions and effects regarding plant development and growth (Marowa et al. 2016). To date, research efforts have been concentrated on elucidating the mode of action and the biological function of expansins belonging to the two largest subfamilies, EXPA and EXPB, and, to a lesser extent EXLA, whilst little attention was given to the EXLB members (Cosgrove 2016; Marowa et al. 2016).

Recently, genome-wide investigations of expansins in species belonging to Fabaceae family, such as *Glycine max* (soybean), *Phaseolus vulgaris* (common bean) and *Medicago truncatula* (Cosgrove 2015; Liu et al. 2015; Zhu et al. 2014) have brought new insights into the molecular and evolutionary history of expansins in legumes, as well as their functions and regulatory mechanisms. In *G. max*, the expansin superfamily has expanded predominantly by segmental duplication and presents a broad functional divergence among subfamilies (Zhu et al. 2014). Likewise, studies in *M. truncatula* revealed that both segmental and tandem duplications contributed to the evolution and diversification of expansins and that genes involved in same processes are closely located on chromosomes (Liu et al. 2015). In this regard, the recently available genome sequences of *Arachis duranensis* and *A. ipaënsis*, the wild progenitors of cultivated peanut (*A. hypogaea*) (Bertioli et al. 2016; Chen et al. 2016), represents a great opportunity to advance the knowledge on plant expansins in *Arachis* and other legumes.

Wild *Arachis* species have been exploited in the last years as sources of alleles to enhance environmental adaptability and to disclose candidate genes, genetic markers, and genomic sequences for peanut breeding improvement (Brasileiro et al. 2014; Janila et al. 2016). In this context, previous transcriptome analysis revealed several differentially expressed genes responsive to drought, including expansin genes, in *A. duranensis* and *A. magna* (Brasileiro et al. 2015; Guimaraes et al. 2012). Moreover, *A. stenosperma* transcriptome survey identified, amongst other candidates, expansin genes putatively involved in resistance to the root-knot nematode (RKN) *Meloidogyne arenaria* (Guimaraes et al. 2015). Either through harboring improved performance under water-limited conditions (*A. duranensis* and *A. magna)* or higher resistance to several pathogens (*A. stenosperma*), these wild species proved to be valuable for stress-related gene discovery.

The potential of expansin-coding genes to enhance stress tolerance, associated with the limited knowledge of their roles in plant responses to environmental cues, highlights the importance of characterizing the expansin superfamily and their functional mechanisms, in order to enable their use for plant improvement. The present study reports the first genome-wide identification and analysis of the expansin superfamily in the genus *Arachis* and the molecular and functional characterization of a novel stress-responsive expansin-like B gene (*AraEXLB8*), a member of the less-studied expansin subfamily.

## Results

### Genome-wide identification and analysis of *Arachis* expansins

**Fig. 1:**
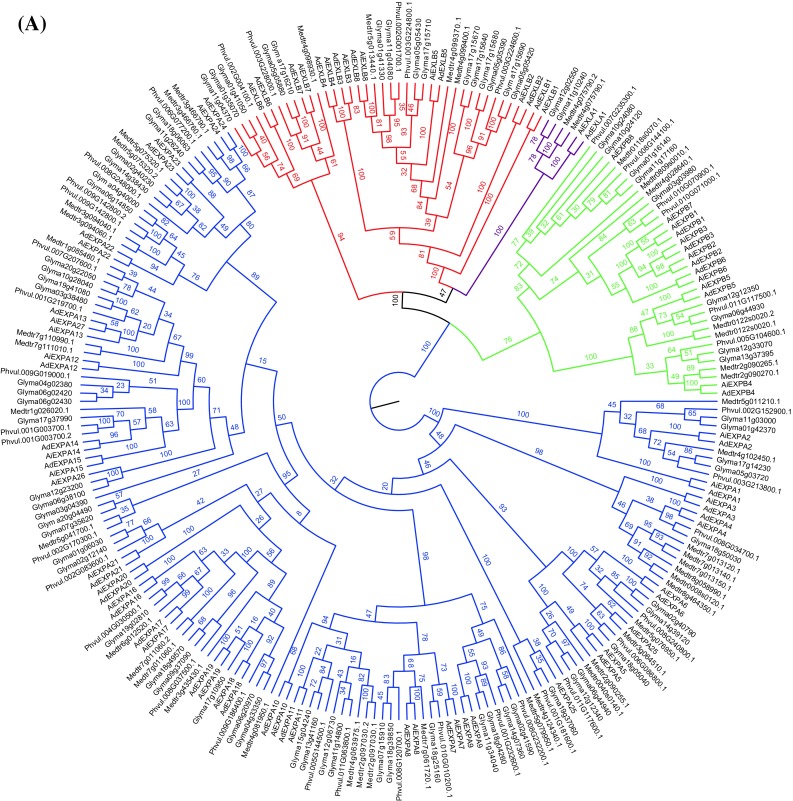

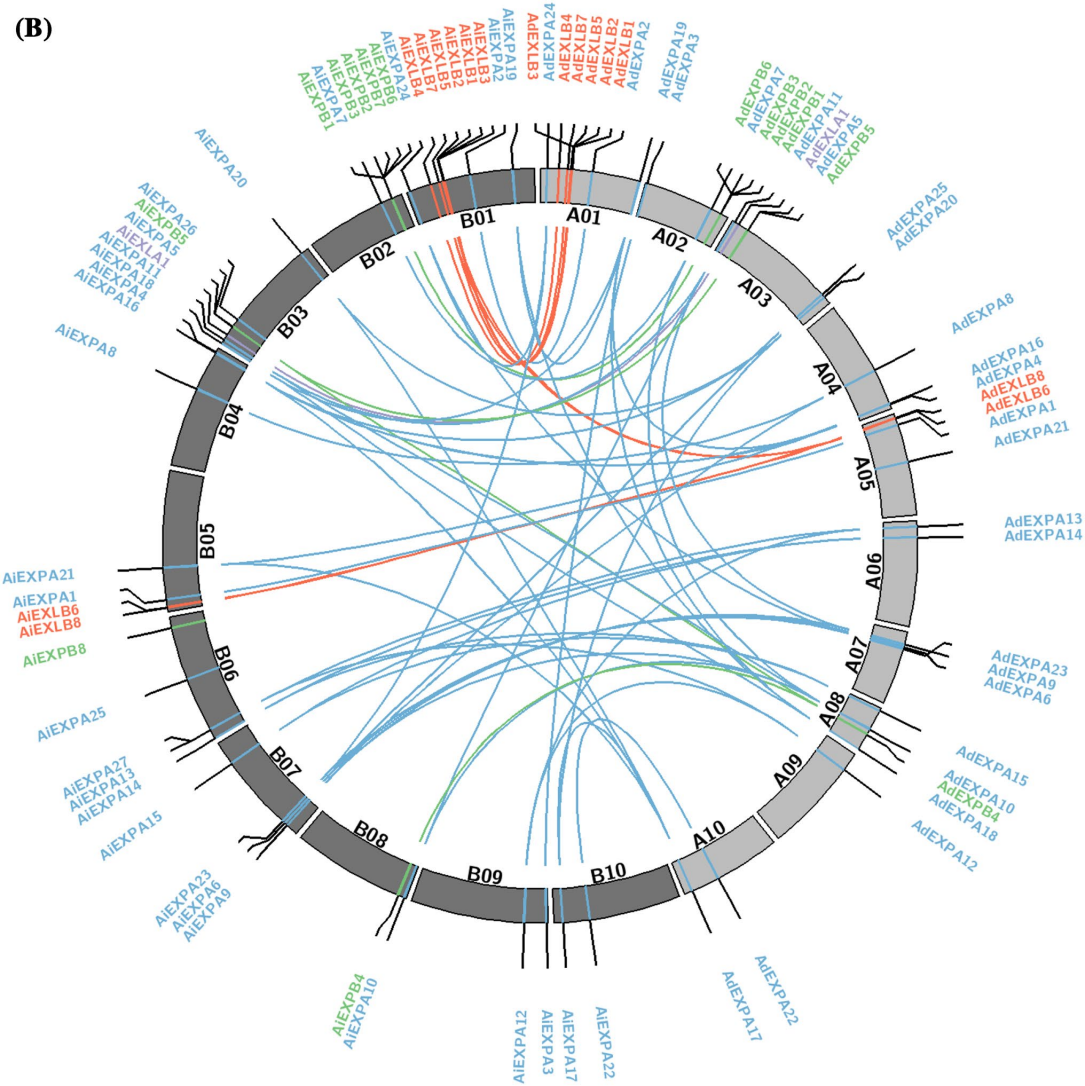
Phylogenetic analysis and expansin distribution in *Arachis duranensis* and *Arachis ipaënsis*. **a** Phylogenetic analysis of the expansin superfamily in *A. duranensis* (Ad); *A. ipaënsis* (Ai); *Glycine max* (Glyma); *Phaseolus. vulgaris* (Phvul) and *Medicago truncatula* (Medtr). **b** Distribution of expansin genes in the ten chromosomes of each *A. duranensis* (*light gray*; A01–A10) and *A. ipaënsis* (*dark gray*; B01–B10). Synteny between the two genomes is represented by *lines*. EXPA (*blue*), EXPB (*green*), EXLA (*purple*) and EXLB (*red*)

Overall, 40 and 44 putative expansins were identified in the genomes of *A. duranensis* and *A. ipaënsis*, respectively, with most of them ranging between 250 and 275 amino acids (AAs) in size and harboring signal peptides for secretion (Supplementary Tables 1 and 2), as expected from canonical plant expansins (Sampedro and Cosgrove 2005). Using a phylogenetic analysis, most of the *Arachis* expansin genes could be assigned to the four subfamilies proposed by Kende et al. (2004) with high confidence values (Fig. 1a; Supplementary Figs. 1, 2, 3). The phylogeny showed that EXPA, EXPB and EXL (A and B) subfamilies formed highly supported monophyletic groups. However, within the EXL group, EXLB proteins did not form a highly supported monophyletic group but were interspersed by the highly supported EXLA monophyletic group, although this separation of the EXLBs in two groups received only a moderate bootstrap support value of 47 (Fig. 1a). A further phylogenetic analysis, including only EXLAs and EXLBs, confirmed either the separation of EXLB in two subgroups (Supplementary Figs. 4a, 5) or low support for the monophyly of EXLBs (Supplementary Fig. 4b). Among *A. duranensis* expansins, the EXPA subfamily constituted the largest clade with 25 members, followed by eight EXLB, six EXPB and one EXLA member, whilst for *A. ipaënsis*, expansins were classified as 27 EXPA, eight EXLB, eight EXPB, and a single EXLA (Fig. 1a; Supplementary Tables 1, 2, 3).

Expansins were unevenly distributed on all the chromosomes of both *Arachis* species (Fig. 1b). Whilst the EXPA members (blue) were distributed on each chromosome of both species, EXLB (red) and EXPB (green) tend to be grouped on chromosomes 1 and 2, respectively, and appear to be mutually exclusive in their locations in each species. Similar uneven distribution of the EXLB members, clustered in one or two chromosomes, was previously observed in *Malus* x *Domestica* (Zhang et al. 2014b) and *G. max* (Zhu et al. 2014).

Whole genome level analysis showed that the majority of the predicted protein-coding genes for *A. duranensis* (87.2%) and *A. ipaënsis* (88.6%) were duplicated. In both genomes, these duplicated copies resulted mostly from dispersed gene duplication (>68%) all around the genomes, whilst less than 17% resulted from whole genome duplication (WGD) (Supplementary Table 4). In contrast, expansion of the expansin superfamily genes mainly resulted (>50%) from WGD/segmental duplication events in both species rather than from dispersed duplications (Supplementary Table 7). Within the expansin subfamilies (Supplementary Table 5), the most common type of duplication for EXPA and EXLB was WGD/segmental duplication, whilst for EXPB, tandem duplications represented the majority. Further analysis also showed that the expansion of the expansin subfamilies in *Arachis* was comparable to that observed in *Arabidopsis thaliana* and *G. max* (Supplementary Table 5).

To predict the selective pressure between *Arachis* expansins duplicated genes, the ratio of *Ka* (rate of non-synonymous mutations)/*Ks* (rate of synonymous mutations) was calculated and revealed that only the *AiEXLB5*/*AiEXLB8* genes pair from *A. ipaënsis* appeared to have undergone positive selection (*Ka*/*Ks* ratio > 1).

Phylogenetic analysis indicated that most duplication events of the expansin genes preceded *A. duranensis* and *A. ipaënsis* divergence (Fig. 1a) and might have occurred in a common ancestor, most probably since the divergence of the Dalbergioid clade (Bertioli et al. 2011). Although large inversions of both arms have been previously observed in chromosome 1 of *Arachis* species (Bertioli et al. 2016), most of the conserved syntenic blocks of duplicated expansins are located on chromosomes 1, 2 and 3 (Fig. 1b). Moreover, the high density of expansin genes in *A. duranensis* chromosome 8 is in accordance with the gene-rich characteristic of this abnormally small ‘A chromosome’ that contains low repetitive content and many genes (Bertioli et al. 2016).

### *Arachis* expansin gene and protein structures

**Fig. 2:**
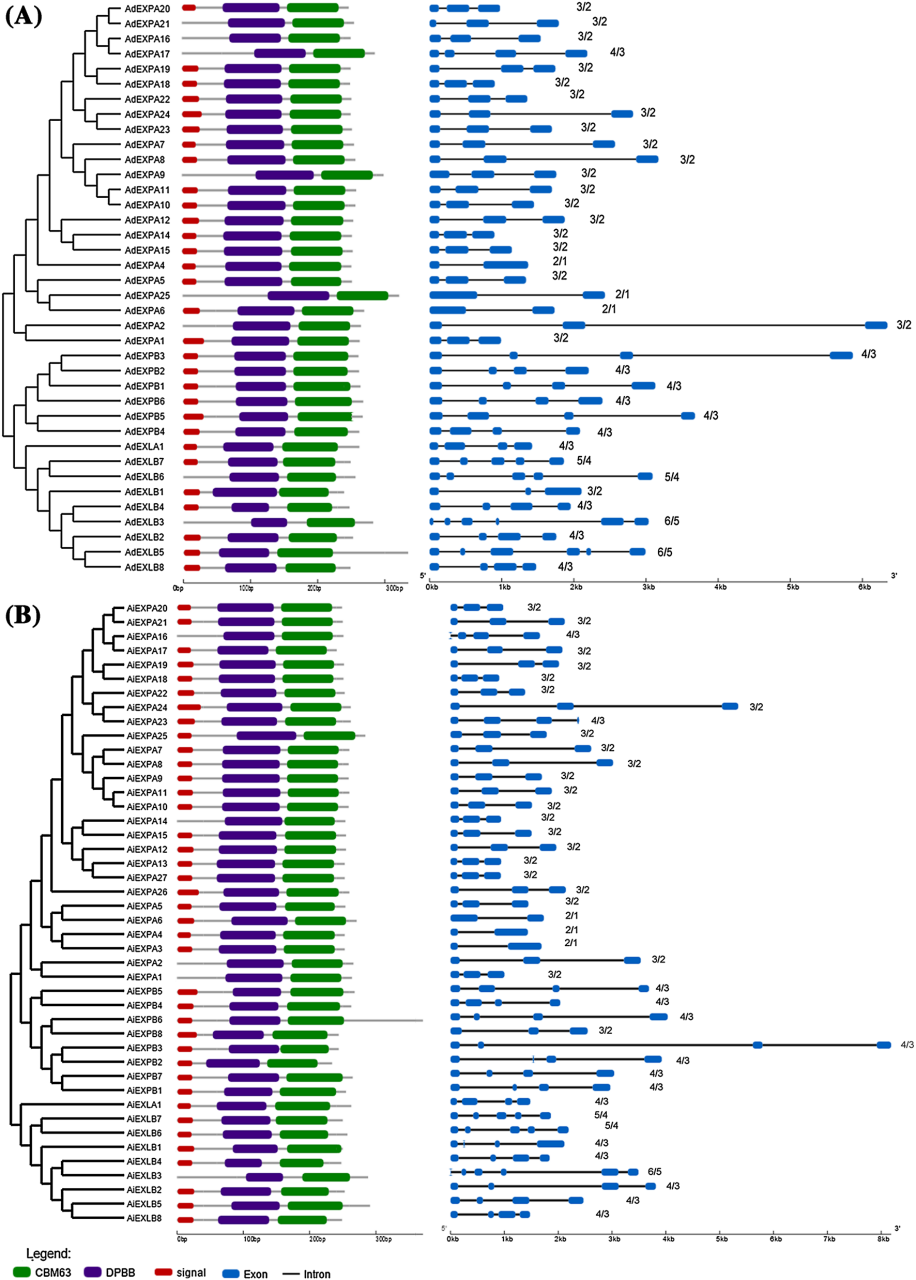
Protein and exon/intron gene structure of expansins in **a** *Arachis duranensis* and **b** *Arachis ipaënsis*. Protein structure diagram (*left*): DPBB and CBM63 domains are represented by *purple* and *green boxes*, respectively, and peptide signals by *red boxes*. Gene structure diagram (*right*): exons are represented by *blue boxes* and introns by linking *single lines*. The number of introns and exons, respectively, is after each diagram

*Arachis duranensis* and *A. ipaënsis* shared conserved expansin gene structures (Fig. 2) and showed diverse organizations comprising of two to six exons. Members of the same phylogenetically-determined subfamily were characterized by a similar intron/exon organization; in particular, those of the EXPB subfamily, in which all members consisted of four exons, except *AiEXPB8*. The majority of the EXPA members (82%) had three exons, while EXLB genes presented the largest number of exons, ranging from four to six, similar to *Malus* x *Domestica* and *G. max* (Zhang et al. 2014b; Zhu et al. 2014). The EXLA members had the same gene structure (4-exon/3-intron) in both *Arachis* species which differs from the usual 5-exon/4-intron EXLA structure of other plants (Ding et al. 2016; Krishnamurthy et al. 2014; Sampedro et al. 2005).

Predicted expansin protein structures (Fig. 2) showed a highly conserved pattern of DPBB and CBM63 Interpro domains (Cosgrove 2015) (Supplementary Figs. 2, 3), preceded by a signal peptide with 21 to 36 AA in most N-terminus. Also, the highly conserved intron/exon structure of *Arachis* expansins within each subfamily supports the classification proposed here, as well as the evidence of the close evolutionary relationship among them. Two EXPA members (*AdEXPA3* and *AdEXPA13*) contained unusual additional copies of DPBB or CBM63 domains in their predicted AA sequences. Since these atypical structures could be the result of gene prediction errors, they were not considered for further characterization.

### RNA-seq expression profiling of expansin genes under biotic and abiotic stresses

**Fig. 3:**
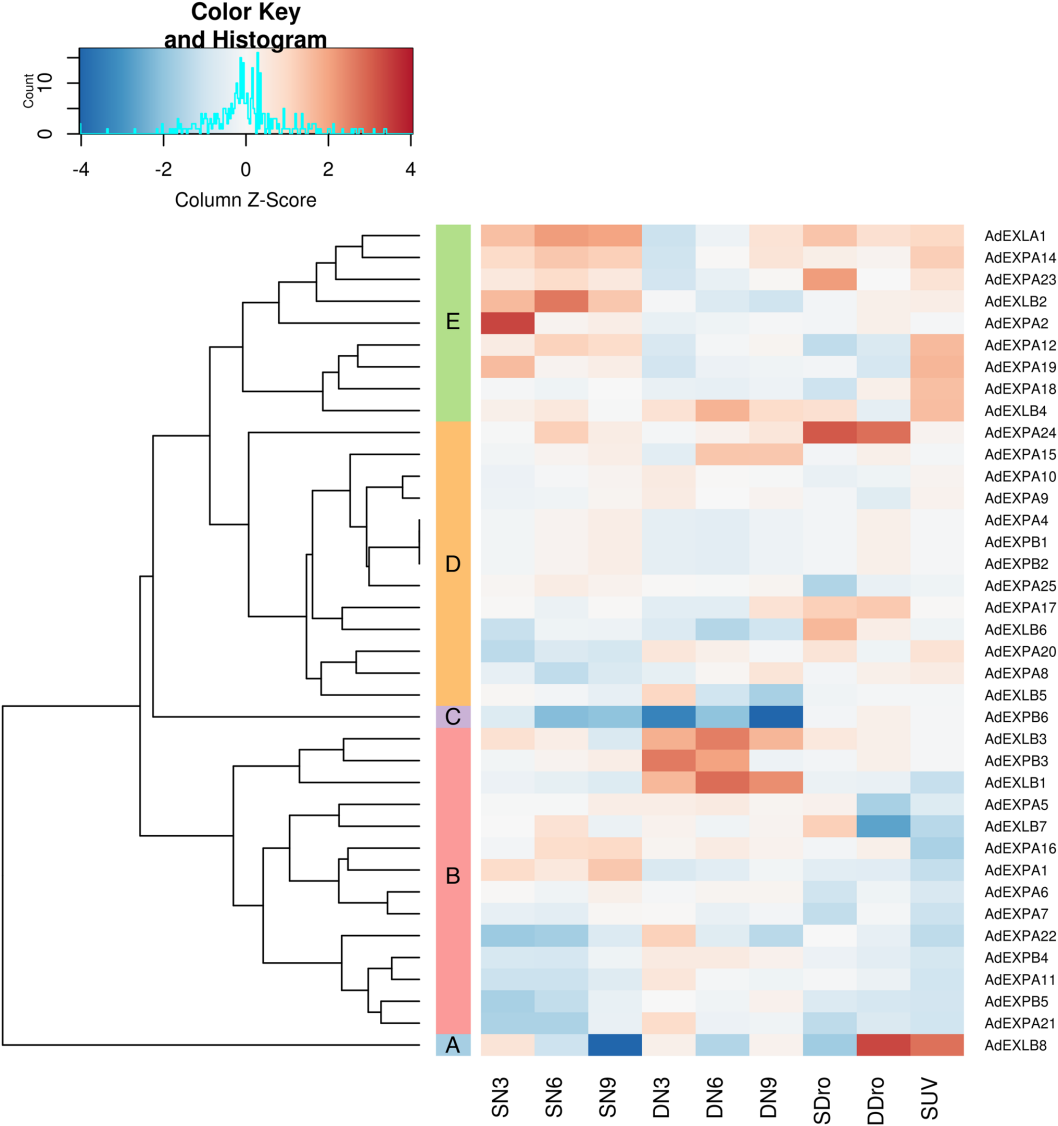
Heatmap of expansin transcripts of *Arachis duranensis* genes and *Arachis stenosperma* orthologs. Expression patterns (log2-based values) in roots of *A. duranensis* (D) expansin genes and *A. stenosperma* orthologs (S) at 3 (N3), 6 (N6) and 9 (N9) DAI with *M. arenaria*, during drought stress (Dro) and after UV exposure (UV), based on RNA-seq data

In order to gain broader understanding of the *Arachis* expansin superfamily gene regulation in response to stress, our previous transcriptome data of *A. duranensis* and *A. stenosperma* plants submitted to abiotic (water deficit and UV exposure) and biotic (RKN *M. arenaria* inoculation) stresses were here exploited. The expression profile (Fig. 3) indicated that all the *Arachis* expansin genes were modulated in response to at least one of the imposed stresses, with distinct expression behaviors depending on the species and stress. The hierarchical clustering analysis classified the expansin genes into five clusters (A–E) according to similarities in their transcript abundance patterns (Fig. 3). Clusters A and C were the most distinct, as each harbored only one representative, *AdEXLB8* and *AdEXPB6*, respectively, as a result of their unique expression profiles. Clusters B and D contained a similar amount of genes (14 and 13, respectively), encompassing representatives of three subfamilies (EXPA; EXPB and EXLB), whereas cluster E, with nine genes, included a single member of EXLA subfamily (*AdEXLA1*) but no representatives of the EXPB subfamily. Genes forming cluster D showed slight variations on their expression levels in response to the variable stresses applied. Differently, higher levels of gene expression and contrasting expression behaviors of *A. duranensis* expansin genes and *A. stenosperma* orthologs were observed between clusters B and E (Fig. 3). In cluster E, the majority of expansins were upregulated in *A. stenosperma* orthologs upon *M. arenaria* inoculation in roots (SN3, SN6 and SN9) or UV exposure in leaves (SUV), whereas a general downregulation was observed in *A. duranensis* (DN3, DN6 and DN9; Fig. 3). Interestingly, in cluster B, the opposite expression behavior occurred in response to *M. arenaria* infection (Fig. 3), in particular for *AdEXLB1, AdEXLB3* and *AdEXPB3* genes, which showed a strong upregulation in *A. duranensis*. Regarding the drought imposition treatment, however, the expression profile displayed by expansins belonging to clusters B, C and E (Fig. 3) were quite similar between *A. duranensis* (DDro) and *A. stenosperma* orthologs (SDro). These results suggest that *Arachis* expansin genes may exhibit both functional differentiation in the two species and overlapping responses between abiotic and biotic stresses.

An expansin-like B gene (*EXLB8*) has drawn attention as it represented an outlier among the *Arachis* expansin genes, with a highly specific expression in response to each stress (Fig. 3). Moreover, our previous studies suggested the involvement of *EXLB8* in RKN-resistance in *A. stenosperma* (Guimaraes et al. 2015), as well as in drought tolerance in *A. duranensis* and *A. magna* (Brasileiro et al. 2015). *EXLB8* gene from wild *Arachis* species (hereafter named *AraEXLB8*) was therefore selected for a broader characterization at molecular and functional levels, as the most promising candidate, among the *Arachis* expansin genes, to be further exploited for plant genetic engineering.

### Characterization and phylogenetic analysis of *AraEXLB8* gene

The 13 *AraEXLB8* coding sequences here identified (NCBI accession numbers KX588113 to KX588125), together with three additional available sequences, were used for the phylogenetic analysis. These 16 *AraEXLB8* genes from different *Arachis* genotypes showed 97.7% of identity, with aligned segment sizes ranging from 740 to 755 bp. Overall, 19 nucleotide substitutions were detected, with the majority (n = 12) occurring in exon 3 (Table 1; Supplementary Tables 6, 7). Based on Blosum62 matrix, most of substitutions can be considered relatively conservative. Although four SNPs (1; 2; 11 and 13) were identified as non-synonymous/non-conservative (Supplementary Tables 6, 7), these nucleotide substitutions did not seem to change protein conformation, as suggested by the *AraEXLB8* 3D protein modeling predicted for four species (*A. duranensis; A. stenosperma; A. ipaënsis* and *A. batizocoi*) which showed no differences among their tertiary structure (data not shown). Interestingly, even without any putative effect on protein folding/structure, SNPs 11 and 13 occurred in the vicinity of a catalytic site presented in the crystal structure of a native *Zea mays* EXPB1 (Yennawar et al. 2006).

**Table 1:** Haplotypes of *AraEXLB8* gene in the 16 *Arachis* genotypes based on coding sequences segment (755 bp)

SNP			1	2	3	4	5	6	7	8	9	10	11	12	13	14	15	16	17	18	19	Genome type			
H	Fs	Fr	E1				E2	E3												E4		A	B	K	AB
H1	5	31	G	C	G	C	C	A	G	A	C	A	A	C	A	A	T	C	C	C	C	–	–	–	5
H2	1	6	*	*	*	*	*	*	*	*	*	*	*	*	*	*	C	*	*	*	*	1	–	–	–
H3	1	6	*	*	T	T	*	*	*	*	*	*	*	*	*	*	C	*	*	*	*	1	–	–	–
H4	1	6	*	T	*	*	*	*	*	*	*	G	*	*	*	G	*	*	*	*	*	–	–	–	1
H5	1	6	*	*	T	*	*	T	*	*	*	*	C	*	*	*	C	G	*	G	T	–	–	1	–
H6	3	19	*	*	T	*	*	*	T	*	G	*	*	*	T	*	C	*	T	G	T	2	1	–	–
H7	1	6	*	*	T	*	G	C	*	T	*	*	C	T	*	*	C	*	*	G	T	–	1	–	–
H8	3	19	A	*	T	*	*	*	*	*	*	*	C	T	*	*	C	*	*	G	T	1	2	–	–

*H* Haplotype; *SNP* single nucleotide polymorphism; *Fs* simple frequency; *Fr* relative frequency (%); *E* exon; * the same base of the first haplotype (H1)

Eight haplotypes were further identified without any recombination event occurring (Table 1). Haplotype 1 (H1) stands out as it comprised all tetraploid genotypes (AB) analyzed here, with the highest SNP frequency being 31%. Other haplotypes included only wild diploids, such as the H6 with two A (*A. villosa* and *A. stenosperma*) and one B (*A. gregoryi*) genome species, while the H8 comprised one A (*A. cardenasii*) and two B (both *A. ipaënsi*s) species.

Phylogenetic analysis conducted with the above 16 *AraEXLB8* coding sequences showed two distinct groups, with a posterior probability support value from 0.52 to 1, based on Bayesian Inference analysis (Supplementary Fig. 6). The first clade comprised all six tetraploids [AB: wild (n = 1), synthetic (n = 1), and cultivated (n = 4)] derived from two distinct genotypes of *A. duranensis* (A genome) whilst the second clade was composed exclusively of diploids species with different genomes (A, B, and K).

### *AraEXLB8* expression in response to stresses

**Fig. 4:**
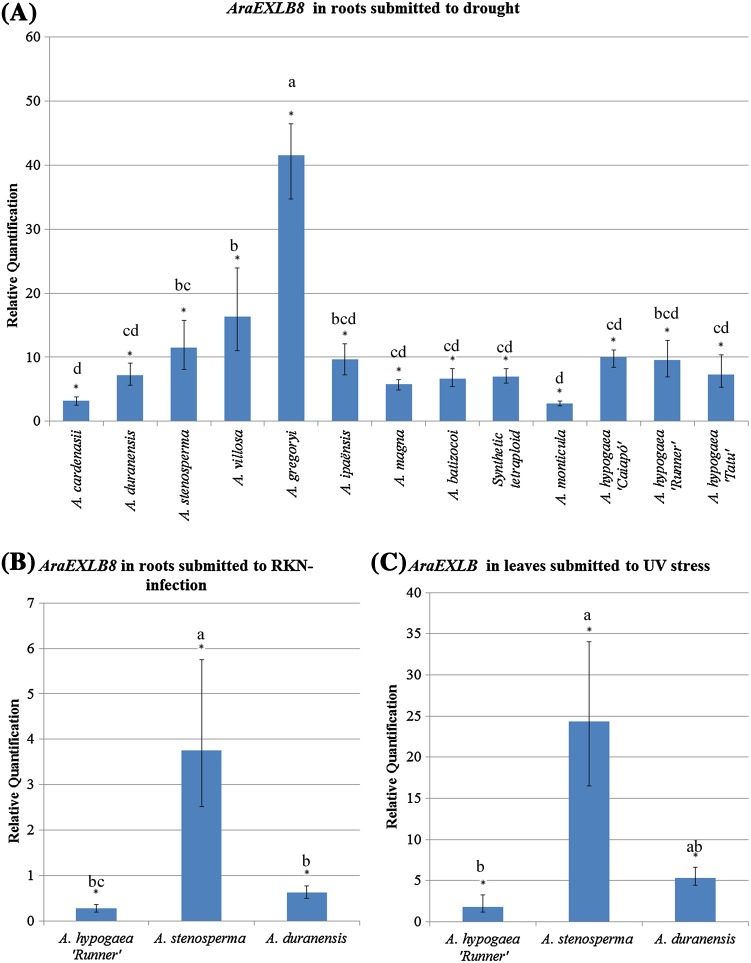
Relative quantification (RQ) of *AraEXLB8* transcripts. Expression profiles of *AraEXLB8* determined by qRT-PCR in **a** roots of 13 *Arachis* genotypes at the NTR around 0.3; in *A. hypogaea* ‘Runner’, *A. duranensis* and A. stenosperma **b** roots inoculated with *M. arenaria* at 3 DAI and **c** leaves 24 h after UV treatment. *Asterisks* significantly regulated genes; *letters* statistical differences between genotype samples

The expression levels of *AraEXLB8* analyzed by qRT-PCR in roots collected at the endpoint of the dry-down assay showed a significant and general upregulation in response to water deficit in all of the 13 genotypes studied (Fig. 4a). Notably, the three wild species with higher expression (*A. gregoryi, A. villosa* and *A. stenosperma* with RQ = 41.5-; 16.3- and 11.5-fold, respectively) are those that were grouped as H6 (Table 1).

Regarding the response to RKN, the *AraEXLB8* expression in roots during early stages of *M. arenaria* infection (3 DAI) displayed a clear contrasting behavior in the three *Arachis* species tested (Fig. 4b): upregulation (3.2-fold) in the RKN-resistant *A. stenosperma* and downregulation in the susceptible *A. hypogaea* ‘Runner’ (0.3-fold) and in the moderately resistant *A. duranensis* (0.6-fold). Despite the current lack of knowledge on the mechanisms through which *AraEXLB8* might operate to contribute to RKN resistance, our results showed a clear trend between *AraEXLB8* expression and plant resistance since the species displaying the lowest expression was the least resistant (*A. hypogaea* ‘Runner’) and the species with the highest expression was the most resistant (*A. stenosperma*). Distinct expression behavior was also observed in response to UV treatment in leaves, where *AraEXLB8* was highly induced in *A. stenosperma* (24.3-fold) but to a lesser extent, in *A. duranensis* and *A. hypogaea* ‘Runner’ (5.3- and 1.8-fold, respectively) (Fig. 4c).

**Fig. 5:**
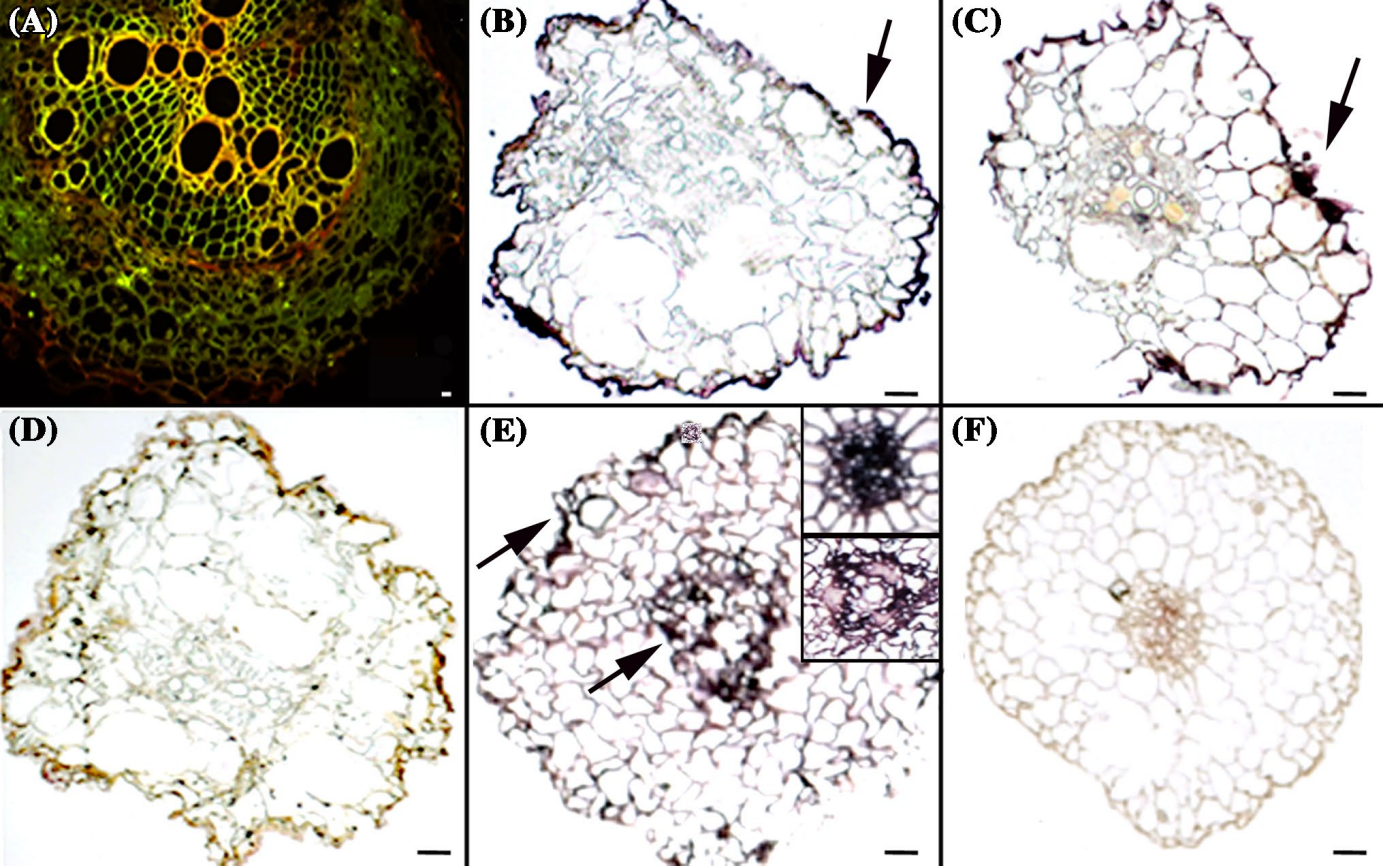
*AraEXLB8 in situ* hybridization. **a** Preserved RNA (*red*) after acridine orange treatment in root cells of a non hybridized RKN-inoculated *A. stenosperma* root (3 DAI). Drought-stressed roots of **b** *A. gregoryi* and **c** *A. villosa* with hybridization signals detected in cortical and epidermal root cells. **d** Well-watered (control) roots of *A. gregoryi*, with no hybridization signals. **e** RKN-inoculated roots showing evident signals in the vascular cylinder and surrounding endodermal cells, as well as some signals in epidermal cells, of *A. stenosperma* (3 DAI). **f** *A. stenosperma* inoculated roots (3 DAI) with no signals after T7 hybridization with the sense (T7) probe (technical control). ISH signals indicated by *arrows. Bars* = 20 µm

To depict the spatial distribution of *AraEXLB8* transcripts in roots, eight *Arachis* genotypes were analyzed by *in situ* hybridization (ISH) using samples collected at the endpoint of the dry-down or few days after *M. arenaria* inoculation. RNA was preserved in all samples as shown by the orange acridine treatment (Fig. 5a). Regardless of the morphological disorganization of the root cells due to drought stress, ISH signals were detected in the outer cortical and epidermal cells, with evident signals in *A. gregoryi* (Fig. 5b) and *A. villosa* (Fig. 5c), and lack of signals in well-watered control roots (Fig. 5d). In RKN-inoculated roots, *AraEXLB8* transcripts were mostly present in the phloem and surrounding endodermal cells, with signals more evident in *A. stenosperma* (Fig. 5e) than in *A. hypogaea* ‘Runner’ (data not shown). No signals were observed in stressed samples hybridized with a sense (T7) probe (Fig. 5f). Hybridization signals showed different intensity depending on the plant genotype and tissue accordingly to the stress imposed.

### Overexpression of *AdEXLB8* in *G. max* composite plants

Three weeks after *Agrobacterium rhizogenes* transformation, *G. max* composite plants developed transgenic hairy roots at the wounding sites (with an efficiency of 78 and 60% with empty and pPZP-AdEXLB8 vectors, respectively), which were inoculated with 1,000 *M. javanica* juveniles. After 60 days, the amount of eGFP-positive roots per composite plant varied between empty and pPZP-AdEXLB8 vectors (36 and 19% of the total hairy roots, respectively) (Supplementary Table 8).

**Fig. 6:**
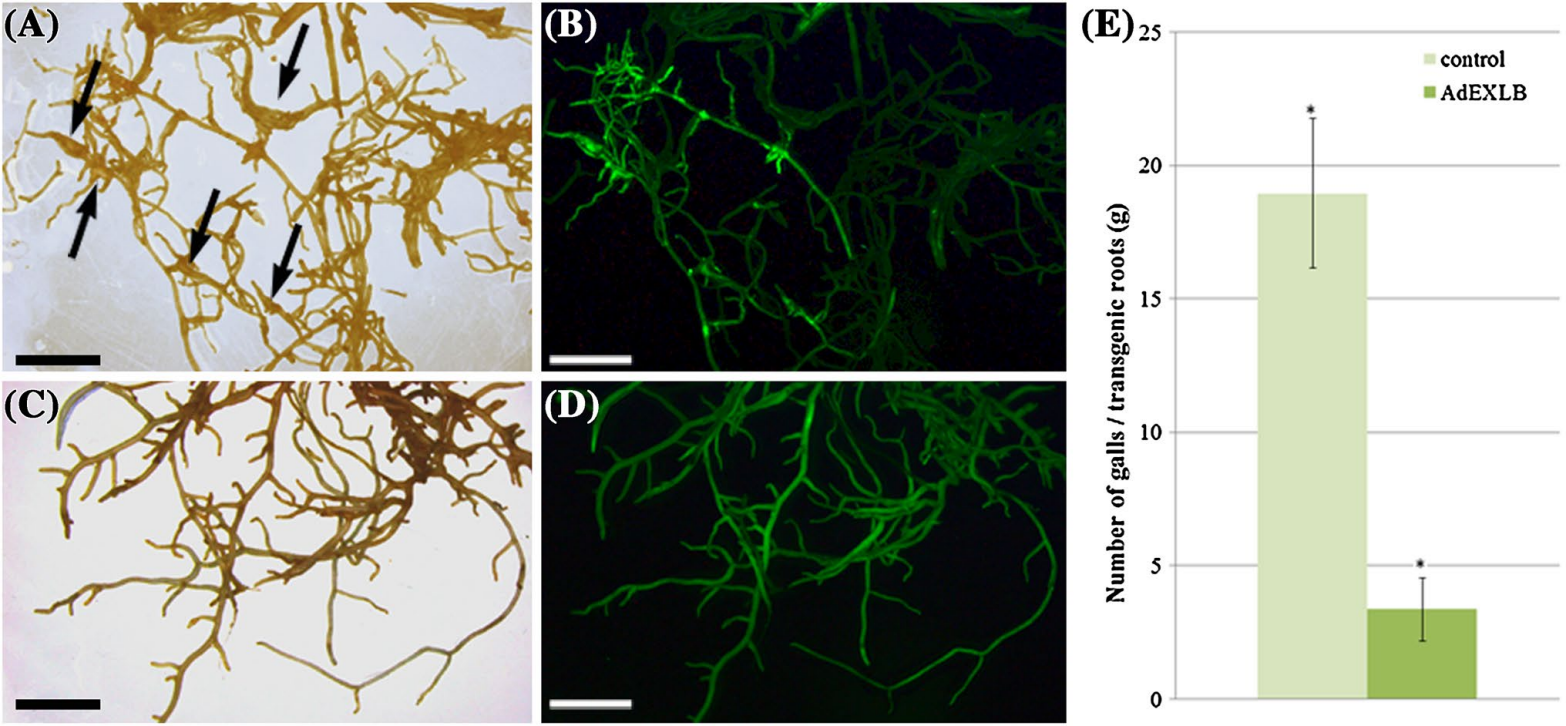
*Glycine max* hairy roots infected with *Meloidogyne javanica* at 60 DAI. **a, b** Hairy roots transformed with empty and **c, d** pPZP-AdEXLB8 binary vectors under **a** and **c** white light and **b** and **d** 480 ± 30 nm excitation GFP filter. **e** Number of galls per gram of hairy roots. Galls are indicated by *arrows. Bars* = 5 mm

*Meloidogyne javanica* juveniles were able to complete at least one full life cycle in *G. max* transgenic hairy roots, as all of the eGFP-positive roots transformed by the empty vector showed gall and egg mass formation, with an average of 19 galls per gram of root (Fig. 6a, b; Supplementary Table 8). Conversely, galls were poorly observed in the eGFP-positive roots transformed with pPZP-AdEXLB8 (average of 3 galls per gram of root), which constitutes a significant reduction of 82% in the number of galls compared to the empty vector control, with results varying for different transformation events (Fig. 6c–e; Supplementary Table 8).

## Discussion

Expansin induction as a preliminary step towards plant adaptation to environmental constraints and pathogen attack has been suggested as one of the mechanisms underlying plant resilience to overcome such stresses (Marowa et al. 2016). Despite their potential role in enhancing plant performance under adverse conditions, molecular and functional characterization of the expansin genes remains incomplete for most species. In this study, expansin superfamily organization, expression dynamics, and evolution was investigated in wild *Arachis* species known for harboring resistance to a large range of biotic and abiotic stresses (Bertioli et al. 2011). Their involvement in responses to drought, nematode infection, and UV treatment is further supported by current results.

### WGD contributed to the expansion of *Arachis* expansins

The number of expansin genes herein identified in *A. duranensis* (40) and *A. ipaënsis* (44) was consistent with that previously described for other legumes which share a genome duplication with *Arachis* at the estimated time of Papilionoid origin (Shirasawa et al. 2013), as *M. truncatula* (42), *P. vulgaris* (36) and *G. max* (75). The higher number of expansin genes in *G. max* may reflect its later lineage-specific duplication. *Arachis* expansins could be further classified into the four subfamilies as proposed by (Kende et al. 2004). However, while EXPA, EXPB and EXLA formed highly supported monophyletic groups, the monophyly of EXLB was not highly supported and EXLBs were separated into two groups interspersed by EXLAs in the main phylogenetic analysis (Fig. 1a). Interestingly, a similar nonsupport of EXLB monophyly and a separation in two subgroups was also observed in a recent Bayesian phylogeny including *Amborella, Arabidopsis* and *Oryza* (Seader et al. 2016). However, this separation of EXLBs in two groups is only supported by low bootstrap (Fig. 1a) or posterior probability (Seader et al. 2016) values. Other phylogenies, including different species, found a monophyly of EXLBs and a separation between EXLAs and EXLBs (Dal Santo et al. 2013; Sampedro et al. 2005; Zhang et al. 2014b; Zhu et al. 2014). However, these topologies either received only moderate support for the monophyly of EXLB (e.g. Supplementary Figs. 1, 4b) or received higher support but either included less representatives of the EXLB family (Sampedro et al. 2005) or made use of less sophisticated phylogenetic methods, known to be more prone to biases such as long branch attraction (Dal Santo et al. 2013; Sampedro et al. 2006; Zhu et al. 2014). Overall, these different phylogenies cast doubt on the support for a monophyly (or polyphyly) of EXLBs, which depends on the taxon sampling and on the phylogenetic method used. However, all the phylogenies converge in supporting with high confidence values, the grouping of all EXLs together and the monophyly of all the other families.

The largely duplicated nature of plant genomes was no exception in *A. duranensis* and *A. ipaënsis*, with current whole genome analysis showing that the majority of protein-coding genes are duplicated, consistent with (Bertioli et al. 2016). The expansion of the *Arachis* expansin superfamily seemed to be mainly achieved by the retention of gene copies after whole genome duplication, as observed for *G. max* and *A. thaliana* (Zhu et al. 2014), or triplication, as for *Brassica rapa* (Krishnamurthy et al. 2014). Differently, in the monocot *Oryza sativa*, tandem duplication was the predominant contributor with a major influence in expansin superfamily expansion (Sampedro et al. 2005; Zhu et al. 2014). Hence, present results support previous evidence that the expansion of the plant expansin superfamily is lineage-specific, and is mostly due to whole genome duplications in eudicots and tandem duplication in monocots (Sampedro et al. 2006). Retention and diversification of gene copies after duplications is one of the main forces in the evolution of higher plants (Freeling 2009; Rodgers-Melnick et al. 2012) and could be associated with subfunctionalization or neofunctionalization of expansin gene duplicates in angiosperms, resulting, for example, in differences in cell wall composition of grasses compared with eudicots (Cosgrove 2015; Sampedro et al. 2015).

In the *Arachis* expansin subfamily, it seems that the abundance of EXPA genes, distributed in every chromosome of both *Arachis* species, might be a result of multiple duplication events, with WGD being the major contributor for their abundance. Conversely, the majority of EXPB gene copies derived from tandem duplications. These duplications were probably responsible for the large EXPB cluster observed in chromosomes 2 of both species. In general, tandem duplications are expected to have a lower impact on the genome than WGD that initially doubles the number of all genes. The EXLB subfamily appears to have expanded specifically in legume species, ranging from 4 to 15 members per genome, whilst in other eudicots, they range from 1 to 4 (Supplementary Table 3). Moreover, in monocots, EXLB duplication showed a different evolution pattern, lacking in *Zea mays* (Zhang et al. 2014c) and having only one representative in *O. sativa* (Sampedro et al. 2005). Herein, the results showed that 50% of the EXLB genes were assigned to the WGD category of duplication. Actually, the EXLB that are mainly concentrated on chromosome 1 are part of a long block of duplicated genes conserved with chromosome 5. The remnant of this WGD are the couples of duplicates *EXLB5* and *EXLB7* on chromosome 1 that correspond to *EXLB8* and *EXLB6* on chromosome 5. This observation holds true for both *Arachis* species. The following series of events for the expansion of EXLBs in *Arachis* can thus be hypothesized: first an ancestral series of proximal or tandem duplications gave rise to an ancestral cluster of EXLBs. Then following a WGD event all these EXLBs were initially duplicated and distributed to the ancestors of chromosome 1 and 5. Then, unbalanced gene loss led to preferential retention on chromosome 1. This phenomenon of genome dominance after WGD leading to more genes retained on one of the duplicated regions has been frequently observed in plants (Murat et al. 2014). Overall, the higher abundance of EXLB genes in *Arachis* suggests a selective advantage to retain these multiple copies after a series of duplication. This implies that the EXLB genes could be part of wild *Arachis* mechanisms that ensure successful adaptability in their native adverse environments (Rodgers-Melnick et al. 2012). Consistent with the possible recruitment of EXLB for new functions, a strong positive selection was identified between *AiEXLB5*/*AiEXLB8* gene copies, suggesting a functional divergence of this duplicated pair over the evolutionary history of (*A*) *ipaënsis* genome. The influence of positive selection in the expansin gene superfamily evolution has previously been reported for *G. max*, but not for other angiosperms such as (*B*) *rapa* or *Z. mays* (Krishnamurthy et al. 2014; Zhang et al. 2014c). This reinforces the present findings that *AraEXLB5* and *AraEXLB8* are stress-responsive genes (Brasileiro et al. 2015; Guimaraes et al. 2015), as positive selection is often observed in genes involved in defense/immunity and response to environmental constraints (Chen et al. 2010).

### *Arachis* expansin genes are widely modulated by stresses

Genome-wide expression analysis demonstrated that expansin genes, regardless of the subfamily, were broadly expressed in roots of *A. duranensis* and *A. stenosperma*, and in leaves of *A. stenosperma*, and were widely responsive to external stimuli, thus confirming their involvement in stress responses. This expansin ubiquitous expression in roots and leaves was previously observed in many plant species, including legumes (Liu et al. 2015; Zhu et al. 2014), which is consistent with their role in root and root hair development, water and nutrient uptake (Che et al. 2016; Cho and Cosgrove 2004) and leaf growth and stomatal plasticity (Lü et al. 2013; Zhang et al. 2011). Moreover, the recently published *A. hypogaea* expression atlas based on 22 different tissue types and ontogenies (Clevenger et al. 2016) showed that 36 homologs out of the 38 *A. duranensis* expansin genes are expressed (absolute expression above 1) in at least one of the tissues that represent the full development (vegetative, reproductive and seed) of peanut. Some of these expansin genes also displayed preferential tissue expression (Clevenger et al. 2016): two *AdEXPB* genes were exclusively expressed in roots or in reproductive shoot tips, whilst five *AdEXLB* transcripts were predominantly located in nodules. This indicates that, although expansins are implicated in a variety of plant processes, some *Arachis* genes might have evolved towards tissue-dependent functional specialization, as suggested for other species (Clevenger et al. 2016; Dal Santo et al. 2013; Lu et al. 2015; Zhang et al. 2014c). As the expression pattern of a gene is usually closely associated with its function, it is assumed that there are species-specific and/or stress-specific features in the biological functions of the *Arachis* expansin genes.

The involvement of expansins in adaptive responses to abiotic stresses has been widely reported and usually associated with the control of cell water loss and osmotic adjustment, probably by promoting cell wall extensibility, or with the general ROS-scavenging defense response to avoid oxidative damage (Marowa et al. 2016; Sasidharan et al. 2011). Here, the majority of the expansin genes were modulated according to the *Arachis* species and stress (drought and UV). Recently, Lu et al. (2015) also demonstrated that the responsiveness of *Solanum lycopersicum* expansins differs depending on the duration and type of stress imposed (drought, cold or heat). The combined evidence implies that different expansins could have distinct functions in response to abiotic stress via an ABA-independent pathway, as previously suggested (Cho and Cosgrove 2004).

Likewise, the overall expression levels of expansin transcripts upon a *M. arenaria* RKN infection revealed a general opposite profile in the RKN-resistant *A. stenosperma* and in the moderately RKN-resistant *A. duranensis*. This might reflect different mechanisms of resistance displayed by these species, which triggers the HR exclusively in *A. stenosperma* and an unknown defense response conferring partial resistance in *A. duranensis* (Proite et al. 2008). Further knowledge about the involvement of expansins in this parasitic association must, therefore, be pursued.

### *AraEXLB8* is a promising molecular marker

Among the *A. duranensis* expansin genes, *AdEXLB8* exhibited a distinct and highly specific expression profile in response to both stresses, corroborating its previously association with *Arachis* drought tolerance (Brasileiro et al. 2015; Ding et al. 2014) and RKN resistance (Guimaraes et al. 2015; Tirumalaraju et al. 2011). *AraEXLB8* nucleotide characterization evidenced the synonymous SNP15 as a promising molecular marker since it occurred exclusively on tetraploid genotypes (natural and synthetic). As previously suggested by Barkley et al. (2007), this type of polymorphism can be further exploited to design new molecular markers to distinguish cultivated tetraploids and wild diploids species in botanical and evolutionary studies. The haplotypes established here based on the *AraEXLB8* nucleotide diversity indicated that they do not share ploidy level (diploidy and tetraploidy), plant life cycle (annual and perennial) or genome type (A, B, D and K), and do not necessarily reinforce phylogenetic proximities, as previously observed for *A. magna* and *A. ipaënsis* (Bechara et al. 2010; Krapovickas and Gregory 1994).

The *AraEXLB8* gene conservation based on cDNA sequences was higher (97.5%) than that observed in previous studies conducted with conserved regions or larger sampling of *Arachis* sequences (Bechara et al. 2010; Cunha et al. 2008; Moretzsohn et al. 2013), reinforcing the close phylogeny for the genotypes here observed. The high levels of conservation on *AraEXLB8* cDNA sequences might reflect *Arachis* reproductive habits, such as autogamy and geocarpy that limit its crossability and seed dispersion (Bertioli et al. 2011; Krapovickas and Gregory 1994).

### *AraEXLB8* is a promising candidate for plant genetic engineering

The cDNA substitution characterizing the haplotype 6 (H6) can be further associated with the *AraEXLB8* expression levels since the three H6 genotypes (*A. stenosperma, A. villosa*, and *A. gregoryi*) displayed the highest induction in response to drought. This overall induction of *AraEXLB8* is coincident with the progressive decline in transpiration previously observed in wild *Arachis* species during a dry-down assay (Leal-Bertioli et al. 2012). Given that expansins are involved in root water uptake and growth (Lü et al. 2013; Zhang et al. 2011), it is possible that this protein, via root-to-leaf signaling, is implicated in both drought response mechanisms triggered upon the perception in roots of soil water availability restriction. Moreover, *in situ* detection of the *AraEXLB8* transcripts indicates cell- and tissue-specific distribution, mostly in cortical and epidermal root cells, the primary site of water decrease perception. These cells might undergo morphology changes to avoid primary tissue damage by increasing expression of drought-responsive genes, such as *AraEXLB8*, involved in perception and signaling of this stress.

Several studies associate the *EXPA, EXPB* and *EXLA* genes with drought perception and adaptation (Marowa et al. 2016), but few reports have identified *EXLB* as drought-responsive genes. Previously, our group reported that *AraEXLB8* is highly upregulated in response to water deficit in roots and leaves of *A. duranensis* and *A. magna* (Brasileiro et al. 2015) with its induction considered to be a preliminary step toward drought acclimation through adjusting cell wall extensibility (Harb et al. 2010). The responsiveness of *AraEXLB8* orthologs to water deficit was also observed in roots of a drought-tolerant *A. hypogaea* genotype (Ding et al. 2014) and other legumes species, *M. truncatula* (Zhang et al. 2014a). Here, the increased expression levels of *AraEXLB8* in the 13 *Arachis* genotypes studied, particularly in those belonging to the H6 group, associated with its specific distribution in surface cells of the roots, suggest that *AraEXLB8* is involved in the network of molecular responses triggered by plants to circumvent dehydration damaging effects, making this gene a promising candidate for drought tolerance engineering.

Additional expression analysis in response to nematode infection in roots and UV treatment in leaves reinforce the prompt *AraEXLB8* responsiveness to other stresses, with different levels of expression displayed by the *Arachis* genotypes that harbor different levels of pathogens resistance. The upregulation of *AraEXLB8* orthologs in response to nematode infection was also reported in the *Arachis* RKN-resistant genotypes (Guimaraes et al. 2015; Tirumalaraju et al. 2011) as well as the downregulation of a *P. vulgaris EXLB* gene at the later stages of its compatible interaction with *M. incognita* (Santini et al. 2016). The shift in the spatial distribution of the *AraEXLB8* transcripts in RKN-inoculated *Arachis* roots, comparing to those detected in drought stressed, indicates the precise and stress-dependent regulation of this gene. The presence of *AraEXLB8* in some cells of vascular bundles reinforces its involvement in RKN infection response, as nematodes in the early parasitic stages (J2) are mainly located within the differentiating vascular bundle (Ozalvo et al. 2014). These results suggest that, regardless of the mechanism of resistance displayed, the *AraEXLB8* gene might have a more downstream role in the pathogen triggering response (PTI), and reinforces the need for a further understanding of molecular, biochemical and cellular events that culminate in pathogen resistance. The notable induction of *AraEXLB8* in *A. stenosperma* leaves in response to UV can be also associated with the general ROS-scavenging and signal transduction pathways that triggered changes in the expression of some genes, as expansins, and in the production of secondary metabolites, as resveratrol, to control numerous biological process, including pathogen defense (Baxter et al. 2014; Han et al. 2015; Lopes et al. 2013), and therefore, contributing to the HR resistance mechanism of *A. stenosperma*, as previously suggested (Guimaraes et al. 2010; Morgante et al. 2013). UV irradiation and other physical stresses induce ‘cross-tolerance’ responses that are commonly associated with pathogen defense and reactions to wounding, promoting increased resistance to pathogens (Mintoff et al. 2015).

*In planta* functional validation of *AdEXLB8* was carried out in *G. max* composite plants since *A. hypogaea* stable transformation is currently limited and time-consuming (Brasileiro et al. 2014; Kuma et al. 2015). This approach allowed the successful *AdEXLB8* overexpression in hairy roots, leading to a remarkable reduction in the number of galls produced by *M. javanica*. Like *M. arenaria*, the *M. javanica* RKN causes important damages in host plants, reducing drastically the productivity of many tropical crops, and it is particularly aggressive in *G. max*, the most important commodity in the world (Beneventi et al. 2013). Since each hairy root obtained is an independent transformation event, a relative high number of eGFP-positive events could be analyzed, reinforcing that *G. max* composite plants are an efficient and fast system to validate *Arachis* RKN resistance candidates (Kuma et al. 2015). Moreover, the selection of transgenic events by eGFP fluorescence allowed the assessment of RKN infection uniquely in *AdEXLB8*-expressing roots, increasing the accuracy and speed of the analysis.

The literature is contradicting in terms of expansin roles in plant–microbe interactions, as the suppression of specific expansin genes reduced the infection by either pathogens, including nematodes (Ding et al. 2008; Gal et al. 2006; Marowa et al. 2016), or symbionts, as arbuscular mycorrhizal fungus (Dermatsev et al. 2010), whilst our results suggest that the overexpression of *AraEXLB8* gene mediates RKN defense in *G. max* roots. The extent to which these non-enzymatic proteins act as physical barriers against microbes or as a source of signals to activate defense responses remains unclear, albeit their induction by auxin could be an underlying mechanism that should be considered (Sasidharan et al. 2011). The preferential expression of the *Arachis EXLB* genes in rhizobium-induced nodules (Clevenger et al. 2016) besides their overall induction upon RKN infection may indicated that the *EXLB* genes are common components in the establishment of both parasitism and symbiosis and seem to play an important role in plant–microbe interactions. In fact, besides expansins, it has been demonstrated that rhizobia and RKN interactions with legumes share a significant number of target genes and display similar molecular mechanisms of symbiosis and pathogenesis to facilitate successful colonization (Damiani et al. 2012).

The effectiveness of *AdEXLB8* overexpression to reduce a RKN infection in stable transgenic plants of soybean and other legumes will enable the assessment of its potential to foster crop improvement for the mitigation of both, abiotic and biotic stresses, which is of utmost importance in the face of the predicted climate changes.

## Materials and methods

### Identification of the *Arachis* expansin superfamily and phylogenetic analysis

The protein sequences of *A. thaliana* and *G. max* expansins (http://www.personal.psu.edu/fsl/ExpCentral) were used to retrieve *A. duranensis* and *A. ipaënsis* expansin sequences from genome assemblies available in PeanutBase (http://peanutbase.org/) Genome Threader software with default parameters (Gremme et al. 2005). Additional sequences were obtained from this database using “expansin” as keyword and TBLASTN searches (e-value 1e−3) against the genomes. The presence of the canonical expansin domains DPBB (PF03330) and CBM63 (PF01357) (Cosgrove 2015) was examined using the CDART tool (http://ncbi.nlm.nih.gov/Structure/lexington/lexington.cgi) and only sequences containing both of the domains were retained.

For phylogenetic analysis, all of the putative *Arachis* expansin protein sequences were aligned using MAFFT software, including sequences from *P. vulgaris, M. truncatula* and *G. max* (https://phytozome.jgi.doe.gov/pz/portal.html) and *Arabidopsis thaliana* (https://www.arabidopsis.org). To provide a more accurate alignment and construction of the phylogenetic tree, sequences with over 90% similarity were trimmed with trimAl (Capella-Gutiérrez et al. 2009). Phylogenic trees were constructed using the Maximum-Likelihood method as implemented in the RAxML software (Stamatakis 2006), with an automatic search of the fittest model and parameters as well as a bootstrap search automatically stopped upon congruence. The nomenclature of *A. duranensis* and *A. ipaënsis* expansins was given according to the synteny between the species and classification in subfamilies accordingly to Kende et al. (2004).

### Genomic distribution, duplication pattern, and structure of expansin genes

The location of the putative expansin genes in *A. duranensis* and *A. ipaënsis* chromosomes was obtained from gene model annotations (http://peanutbase.org/) and all predicted protein sequences were submitted to self and all-against-all BLASTP analyses (e-value 1e− 10). The expansins distribution and respective gene duplication patterns in chromosomes were analyzed by the MCScanX software with default parameters (Wang et al. 2012) and the results were formatted with custom perl script to Circos format (http://circos.ca/) for graphical representation. To detect positive selection between genes, the ratio of *Ka*/*Ks* between the gene copies of expansin sequences was computed using the Nei-Gojobori statistics (Nei and Gojobori 1986), as implemented in McScanX.

*Arachis* expansin gene exon/intron organization retrieved from PeanutBase (http://peanutbase.org/) was submitted to GSDS software (http://gsds.cbi.pku.edu.cn/). InterPro and SignalP were used for protein domain and signal peptide prediction, respectively, and the isoelectric points and molecular weights of the proteins obtained from ExPASy (http://expasy.org/).

### RNA-seq expression profiling of *Arachis* expansin genes

Illumina RNA-seq data previously obtained by our group from roots of *A. duranensis* and *A. stenosperma* plants submitted to drought (available at http://peanutbase.org) or inoculated with RKN *M. arenaria* (collected at 3, 6 or 9 days after inoculation; DAI) (Guimaraes et al. 2015) or from leaves of *A. stenosperma* submitted to UV exposure (unpublished results) were used for the expansin genes expression analysis. The genome of *A. duranensis* was used to create an index with the Kallisto program (version 0.42.3) and only high quality reads from the transcriptome were then used for the quantification against the created index, with default settings (Bray et al. 2016). For the differential expression analysis, the edgeR package (Robinson et al. 2010) was employed. The log2 of fold-change value between control and treated samples in both species was used for the heatmap generation using heatmap2R package (Warnes et al. 2015).

### *AraEXLB8***diversity in***Arachis* genotypes

Total RNA was isolated from young leaves of 13 *Arachis* genotypes (Supplementary Table 9) and the corresponding cDNAs used for RT-PCR as described by Morgante et al. (2011), employing *AraEXLB8-*flanking primers (EXPF 5′-AGTGCTTCATCTCAAAATGGAACT-3′ and EXPR 5′-TCTATTCAAGCTGGATTTGAGTATCA-3′). Sequenced amplification products were aligned using the Sequencher software (Gene Codes, Ann Arbor, USA) with three additional available *AraEXLB8* sequences from *A. duranensis* and *A. ipaënsis* (http://peanutbase.org/gbrowse) and *A. hypogaea* ‘Tifrunner’ [GO326087.1; (http://www.ncbi.nlm.nih.gov)]. Nucleotide substitutions were defined by BLOSUM62 matrix (Henikoff and Henikoff 1992) and hydrophobicity (Kyte and Doolittle 1982) while DNA sequence variation by DnaSP software (http://www.ub.es/dnasp/).

Phylogenetic analysis was conducted using the above 16 *AraEXLB8* coding sequences and four additional outgroups (*G. max* GI:955303815; *P. vulgaris* GI:593787101; *C. arietinum* GI:828330493 and *M. truncatula* Medtr5g013440.1). The phylogeny was constructed by Bayesian Inference using MrBayes program (http://mrbayes.sourceforge.net/) with default parameters and performed with 20 million generations. The phylogenetic model (GTR + I) was selected as the fittest model according to ModelTest and the consensus trees were inspected in FigTree software (http://tree.bio.ed.ac.uk/software/figtree/).

### *AraEXLB8* qRT-PCR analysis and in situ transcripts detection

#### Dry-down assay

Normalized transpiration rates (NTR) were determined for 4-week-old plants of 13 Arachis genotypes (Supplementary Table 9) throughout a soil gradual water deficit assay (dry-down), as previously described (Leal-Bertioli et al. 2012). Roots were collected when each drought stressed (DS) individual reached a NTR value around 0.3 relative to well-watered (WW) controls. Samples from three individuals were assembled forming three independent biological replicates for each treatment (WW and DS), and per genotype.

#### RKN inoculation assay

*Arachis stenosperma, A. duranensis* and *A. hypogaea* ‘Runner’ 4-week-old plants (Supplementary Table 9) were inoculated with *M. arenaria* race 1 at juvenile stage 2 (J2) as described by Morgante et al. (2013). Root samples from five individuals were collected at zero (control), 3 DAI (stressed), and assembled to form two independent biological replicates per treatment and genotype.

#### UV assay

Young leaves were collected from 7-week-old plants of *A. stenosperma, A. duranensis* and *A. hypogaea* ‘Runner’ (Supplementary Table 9) and submitted to UV-C light treatment for 2 h and 30 min, essentially as described by Lopes et al. (2013). Samples from five individuals were collected before (control) and 24 h after (stressed) UV treatment and assembled to form three independent biological replicates for each treatment and genotype.

#### Quantitative RT-PCR (qRT-PCR) analysis

Total RNA was extracted from samples and the cDNA synthesized as previously described (Morgante et al. 2011). The qRT-PCR reactions were conducted in a 7300 Real-Time PCR System (Applied Biosystem) using primers of the *A. duranensis ELXB8* (*AdEXLB8*) gene, as previously described (Brasileiro et al. 2015). In accordance to Morgante et al. (2011), ACT1 and UBI2 were used as reference genes for samples subjected to dry-down whereas 60S and GAPDH to RKN inoculation and UV treatments. The relative quantification (RQ) of transcripts was determined as described by Brasileiro et al. (2015) and statistically tested using one-way ANOVA followed by Tukey’s test.

#### *In situ* hybridization (ISH) analysis

Root fragments were isolated and processed in accordance to (Morgante et al. 2013). A 659 bp cDNA sequence of *AdEXLB8* was amplified (EXPuniF: 5′-ACTGCCAGTCACTTGGAACC-3′ and Exp464R: 5′-AGATCCATTCCGCCATAGC-3′), cloned into pGEM^®^-T Easy (Promega, Madison, USA) and used to produce dig-labeled RNA probes (DIG RNA Labeling Kit SP6/T7, Roche). Slides were then hybridized with 2 ng of probe and hybridization sites were immunocytochemically detected as described (Morgante et al. 2013).

### *Meloidogyne javanica* bioassay in *G. max* composite plants overexpressing *AdEXLB8* gene

The coding region (753 bp) of *AdEXLB8* was cloned (Epoch Life Science, Texas, USA) under the control of the *A. thaliana* actin 2 promoter, at the *XhoI* restriction site of the binary vector pPZP-201BK-EGFP (Chu et al. 2014) containing an enhanced Green Fluorescent Protein (eGFP) to form the pPZP-AdEXLB8 vector. *Agrobacterium rhizogenes* strain ‘K599’, harboring pPZP-201BK-EGFP (empty) or pPZP-AdEXLB8, vectors was then used to generate *G. max* ‘Williams 82’ composite plants employing fresh bacterial paste inoculum (Chu et al. 2014), basically as described before (Kuma et al. 2015). Transgenic roots were identified one week later through eGFP fluorescence under GFP1 filter set on a M205 stereomicroscope (Leica Microsystems, Wetzlar, Germany) and the eGFP-negative roots were excised before plant transfer to a 3:1 sand:plant gel mixture (v:v) and acclimation in greenhouse conditions. Three-week-old composite plants were challenged with approximately 1000 J2 of *M. javanica* and the number of galls determined at 60 DAI. The qRT-PCR analysis of *AdEXLB8* expression in eGFP-positive roots was conducted as described above. Outliers in galls counting data were removed by Grubbs’ test and means compared by unpaired t-test using Graphpad software (http://graphpad.com/quickcalcs/ttest1/).

## Electronic supplementary material

Below is the link to the electronic supplementary material.

Supplementary Fig. 1 Phylogenetic tree of the expansin genes in *Arachis duranensis* (Ad), *Arachis ipaënsis* (Ai), *Glycine max* (Glyma), *Medicago truncatula* (Medtr), *Phaseolus vulgaris* (Phvul) and *Arabidopsis thaliana* (At). The expansin subfamilies are represented by colors: EXPA (*blue*), EXPB (*green*), EXLA (*purple*) and EXLB (*red*). (PDF 56 KB)

Supplementary Fig. 2 Trimmed alignment of the expansin genes of *Arachis duranensis* (Ad), *Arachis ipaënsis* (Ai), *Glycine max* (Glyma), *Medicago truncatula* (Medtr), *Phaseolus vulgaris* (Phvul) and *Arabidopsis thaliana* (At). The tree was constructed with high conserved aminoacids trimmed with trimAl (>90%). The domains DPBB and CBM63 are represented by black and blue dotted lines, respectively. (PDF 8380 KB)

Supplementary Fig. 3 Trimmed alignment of the expansin genes of *Arachis duranensis* (Ad), *Arachis ipaënsis* (Ai), *Glycine max* (Glyma), *Medicago truncatula* (Medtr) and *Phaseolus vulgaris* (Phvul). The tree was constructed with high conserved aminoacids trimmed with trimAl (>90%). The domains DPBB and CBM63 are represented by black and blue dotted lines, respectively. (PDF 7384 KB)

Supplementary Fig. 4 Maximum likelihood phylogenetic tree of the subfamily EXLA and EXLB of *Arachis duranensis* (Ad), *Arachis ipaënsis* (Ai) and *Arabidopsis thaliana* (At). EXLA subfamily is represented in *purple* and EXLB in *red*. Bootstrap support values are indicated on the respective branches. (a) Midpoint-rooted topology, including only EXLA and EXLB subfamilies. (b) Topology rooted on two EXPB sequences (from *Arabidopsis thaliana* and *Glycine max*) in *black*. (PDF 39 KB)

Supplementary Fig. 5 Trimmed alignment of EXLA and EXLB genes of *Arachis duranensis* (Ad), *Arachis ipaënsis* (Ai) and *Arabidopsis thaliana* (At). The tree was constructed with high conserved aminoacids trimmed with trimAl (>90%). The domains DPBB and CBM63 are represented by *black* and *blue* dotted lines, respectively. (PDF 926 KB)

Supplementary Fig. 6 Phylogenetic analysis of *AraEXLB8* genes. The phylogenetic tree was obtained from Bayesian Inference analysis with mean posterior probability support values on branches. Colors indicate *Arachis* genome types [A (*blue*); B (*green*); K (*red*); and AB (*purple*)] and outgroups (*black*). ^1^ = *A. duranensis* accession V14167 (PeanutBase gene identifier number Aradu.MR104); ^2^ = *A. duranensis* accession K7988 (NCBI sequence number KX588115); * = *A. ipaënsis* accession KG30076 (NCBI sequence number KX588120); ** = *A. ipaënsis* accession KG30076 (PeanutBase gene identifier number Araip.J65RE). (DOCX 66 KB)

Supplementary material 7 (XLSX 21 KB)

Supplementary material 8 (XLSX 21 KB)

Supplementary material 9 (DOCX 17 KB)

Supplementary material 10 (DOCX 16 KB)

Supplementary material 11 (DOCX 17 KB)

Supplementary material 12 (DOCX 20 KB)

Supplementary material 13 (DOCX 23 KB)

Supplementary material 14 (DOCX 17 KB)

Supplementary material 15 (DOCX 16 KB)
