# Supplementary figures and images for: Genome-wide analysis of expansin superfamily in wild Arachis discloses a stress-responsive expansin-like B gene
Source: Plant Mol Biol. 2017 Feb 27;94(1):79–96. doi: 10.1007/s11103-017-0594-8 (PMC5437183; doi:10.1007/s11103-017-0594-8)

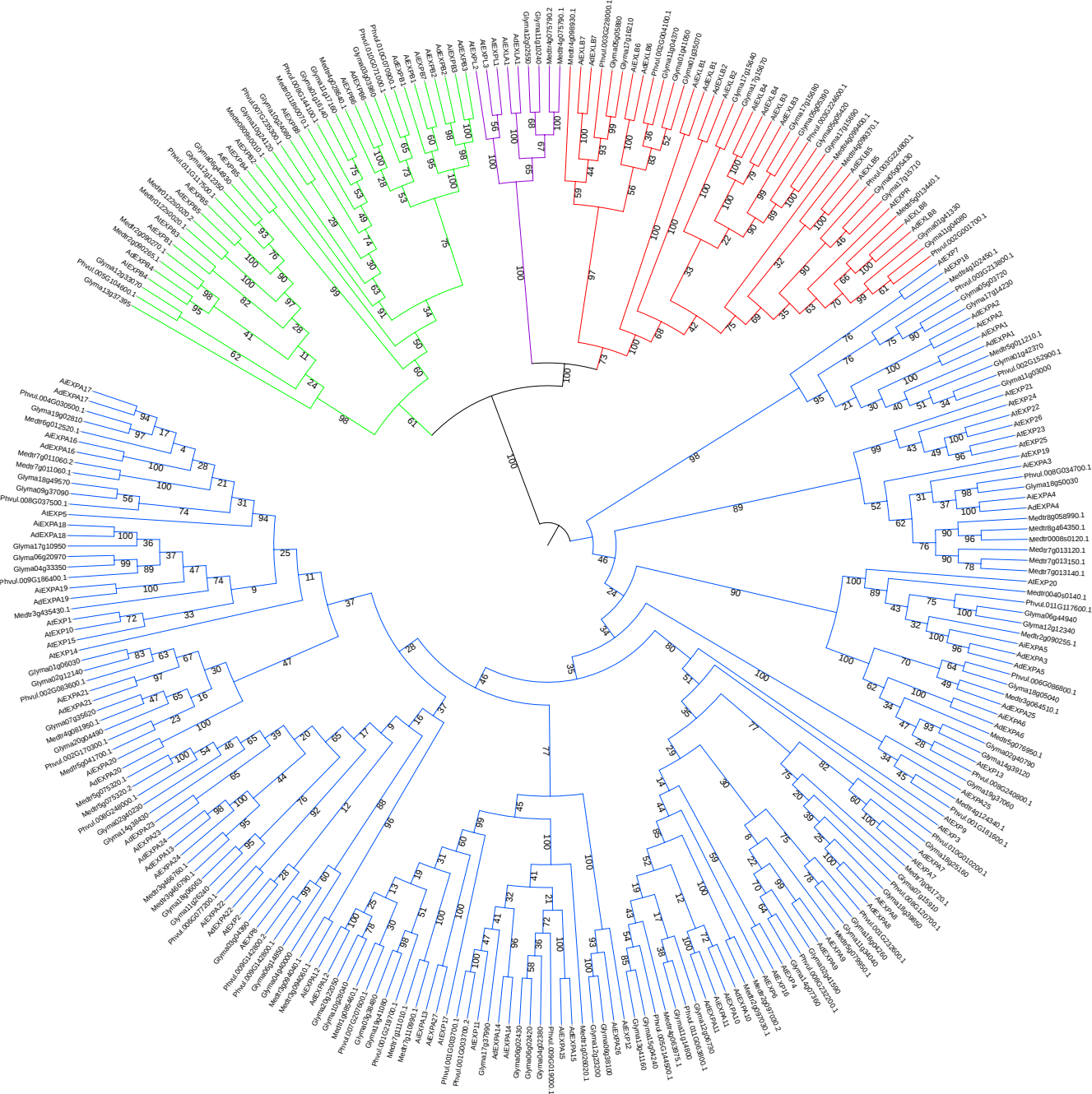

Supplement: Supplementary file 1 — Supplementary Fig. 1 Phylogenetic tree of the expansin genes in Arachis duranensis (Ad), Arachis ipaënsis (Ai), Glycine max (Glyma), Medicago truncatula (Medtr), Phaseolus vulgaris (Phvul) and Arabidopsis thaliana (At). The expansin subfamilies are represented by colors: EXPA (blue), EXPB (green), EXLA (purple) and EXLB (red). (PDF 56 KB) [file 11103_2017_594_MOESM1_ESM.pdf]

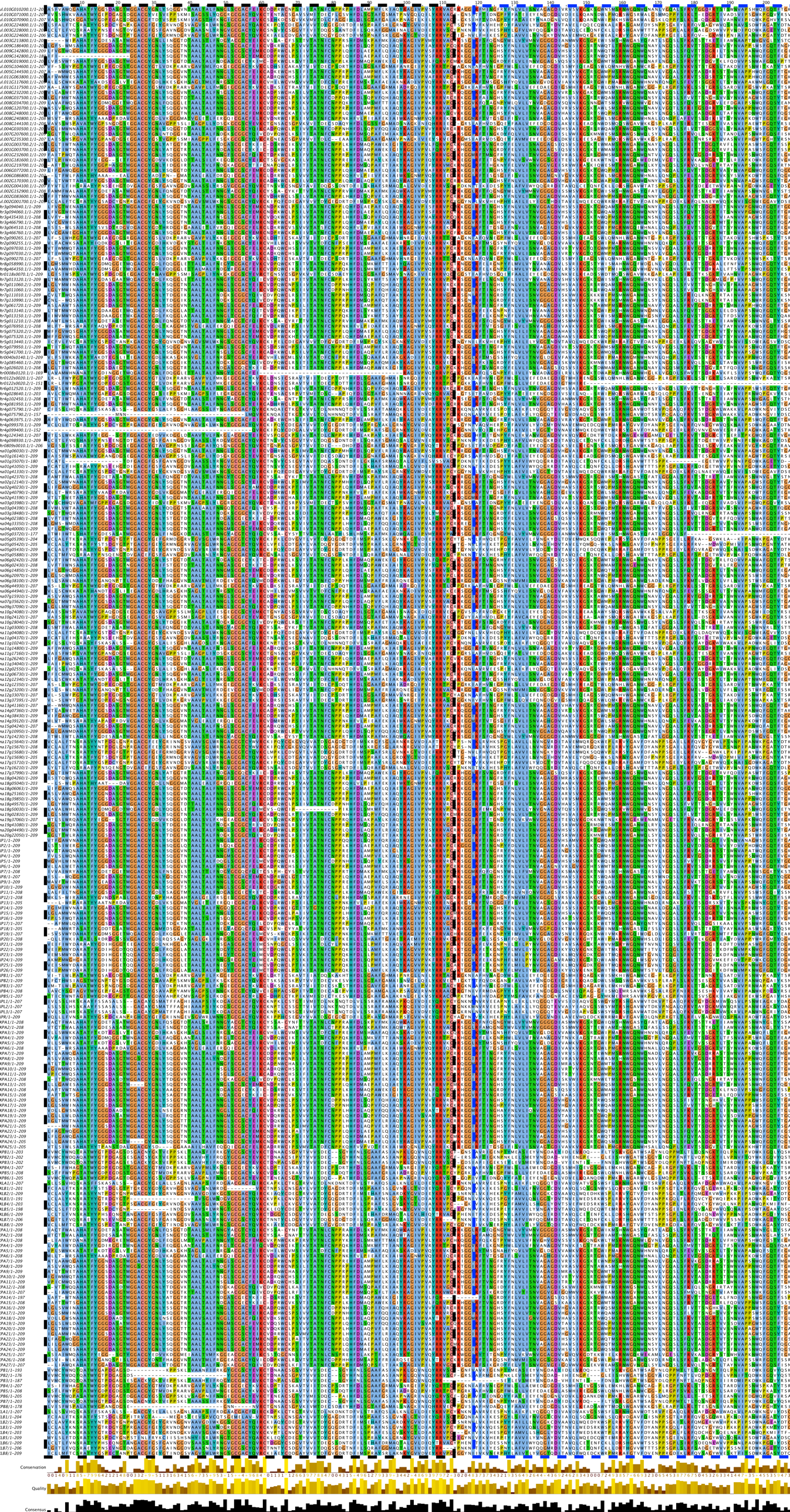

Supplement: Supplementary file 2 — Supplementary Fig. 2 Trimmed alignment of the expansin genes of Arachis duranensis (Ad), Arachis ipaënsis (Ai), Glycine max (Glyma), Medicago truncatula (Medtr), Phaseolus vulgaris (Phvul) and Arabidopsis thaliana (At). The tree was constructed with high conserved aminoacids trimmed with trimAl (>90%). The domains DPBB and CBM63 are represented by black and blue dotted lines, respectively. (PDF 8380 KB) [file 11103_2017_594_MOESM2_ESM.pdf]

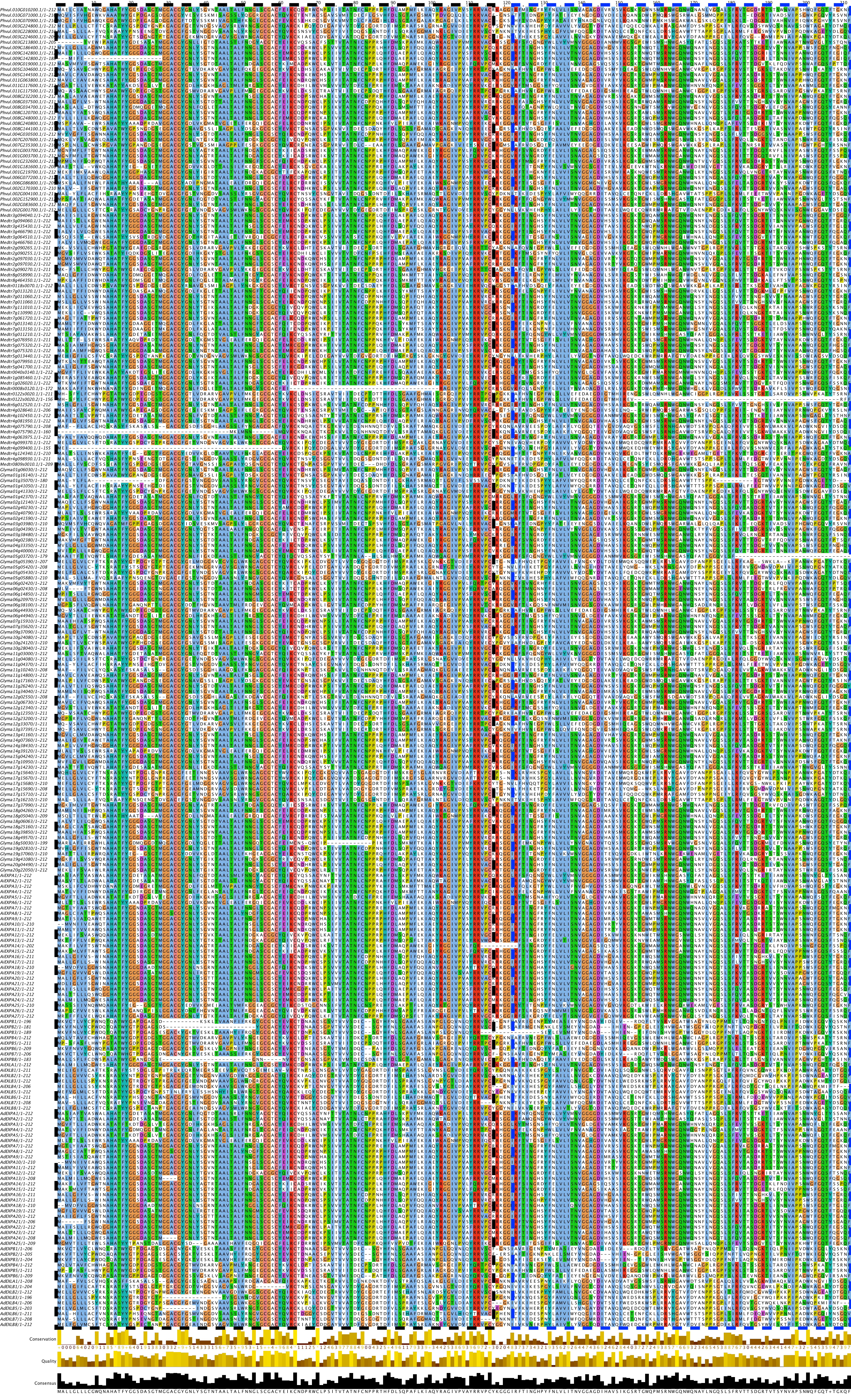

Supplement: Supplementary file 3 — Supplementary Fig. 3 Trimmed alignment of the expansin genes of Arachis duranensis (Ad), Arachis ipaënsis (Ai), Glycine max (Glyma), Medicago truncatula (Medtr) and Phaseolus vulgaris (Phvul). The tree was constructed with high conserved aminoacids trimmed with trimAl (>90%). The domains DPBB and CBM63 are represented by black and blue dotted lines, respectively. (PDF 7384 KB) [file 11103_2017_594_MOESM3_ESM.pdf]

(a)

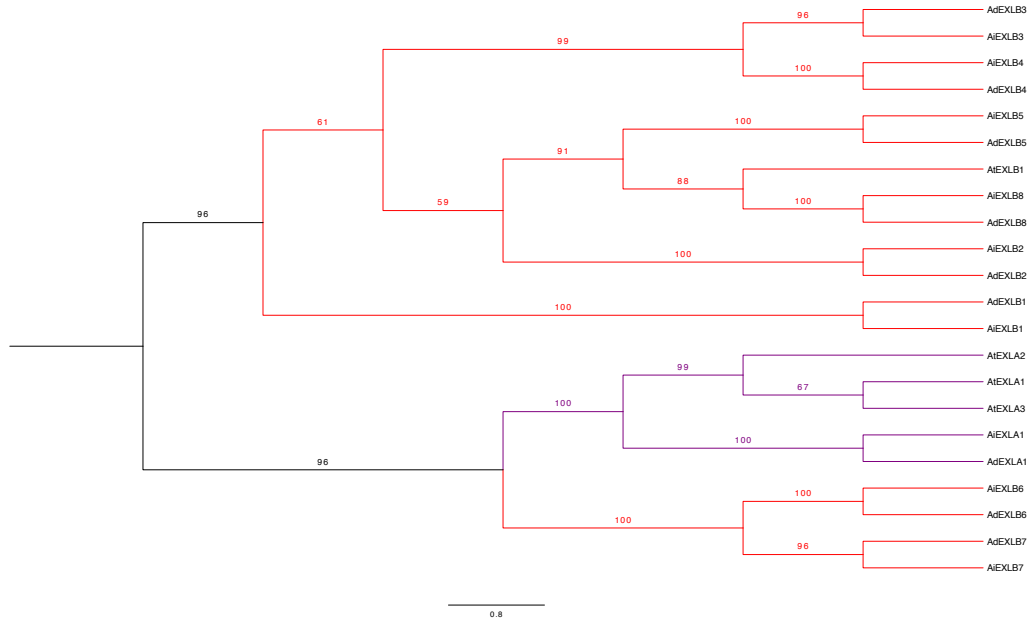

(b)

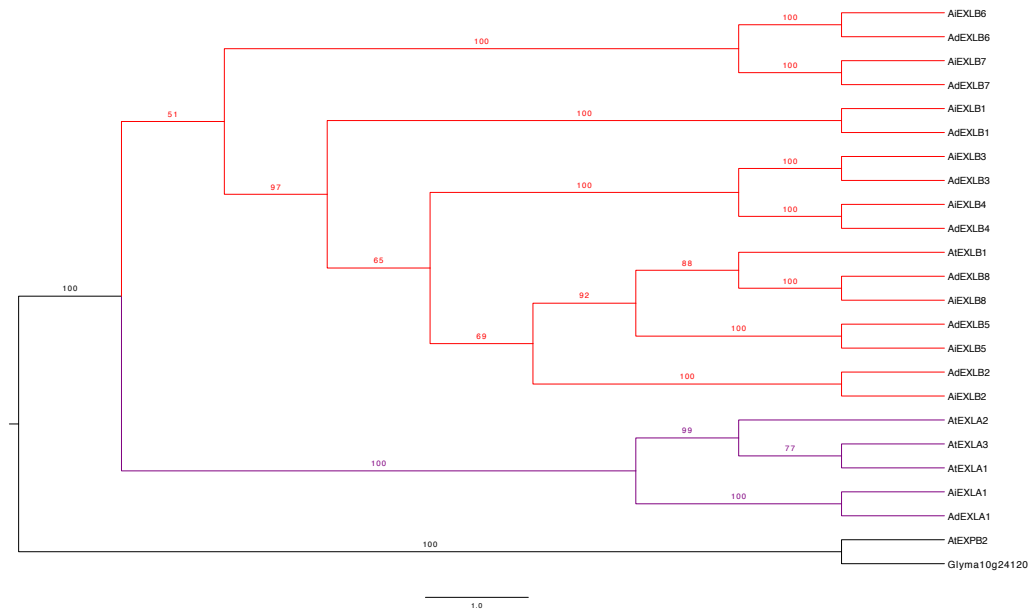

Supplement: Supplementary file 4 — Supplementary Fig. 4 Maximum likelihood phylogenetic tree of the subfamily EXLA and EXLB of Arachis duranensis (Ad), Arachis ipaënsis (Ai) and Arabidopsis thaliana (At). EXLA subfamily is represented in purple and EXLB in red. Bootstrap support values are indicated on the respective branches. (a) Midpoint-rooted topology, including only EXLA and EXLB subfamilies. (b) Topology rooted on two EXPB sequences (from Arabidopsis thaliana and Glycine max) in black. (PDF 39 KB) [file 11103_2017_594_MOESM4_ESM.pdf]

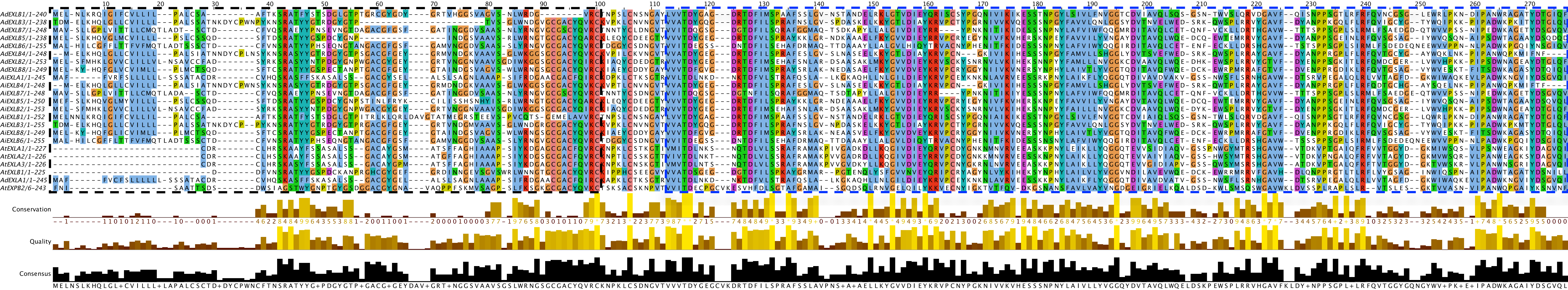

Supplement: Supplementary file 5 — Supplementary Fig. 5 Trimmed alignment of EXLA and EXLB genes of Arachis duranensis (Ad), Arachis ipaënsis (Ai) and Arabidopsis thaliana (At). The tree was constructed with high conserved aminoacids trimmed with trimAl (>90%). The domains DPBB and CBM63 are represented by black and blue dotted lines, respectively. (PDF 926 KB) [file 11103_2017_594_MOESM5_ESM.pdf]
