# Supplementary material for: Genome-wide analysis of expansin superfamily in wild Arachis discloses a stress-responsive expansin-like B gene
Source: Plant Mol Biol. 2017 Feb 27;94(1):79–96. doi: 10.1007/s11103-017-0594-8 (PMC5437183; doi:10.1007/s11103-017-0594-8)
Supplement: Supplementary file 9 — Supplementary material 9 (DOCX 17 KB) [file 11103_2017_594_MOESM9_ESM.docx]

**Supplementary Table 3.** Subfamily distribution of expansin genes in eudicots plant species.

| **Species** | **EXPA** | **EXPB** | **EXLA** | **EXLB** | **TOTAL** | **Reference** |
| --- | --- | --- | --- | --- | --- | --- |
| *Arabidopsis. thaliana* | 26 | 6 | 3 | 1 | 36 | (Sampedro *et al.* 2005) |
| Poplar (*Populus trichocarpa*) | 27 | 3 | 2 | 4 | 36 | (Sampedro *et al.* 2006) |
| Grapevine (*Vitis vinifera*) | 20 | 4 | 1 | 4 | 29 | (Dal Santo *et al.* 2013) |
| Apple (*Malus* × *Domestica*) | 34 | 1 | 2 | 4 | 41 | (Zhang *et al.* 2014b) |
| Chinese cabbage (*Brassica rapa*) | 39 | 9 | 2 | 3 | 53 | (Krishnamurthy *et al.* 2014) |
| Tomato (*Solanum lycopersicum*) | 25 | 8 | 1 | 4 | 38 | (Lu *et al.* 2015) |
| Tobacco (*Nicotiana tabacum*) | 36 | 6 | 3 | 7 | 52 | (Ding et al. 2016) |
| Soybean (*Glycine max*) | 49 | 9 | 2 | 15 | 75 | (Zhu *et al.* 2014) |
| *Medicago truncatula* | 31 | 6 | 1 | 4 | 42 | (Liu *et al.* 2015) |
| Common bean (*Phaseolus. vulgaris*) | 25 | 6 | 0 | 5 | 36 | (Zhu *et al.* 2014) |
| *Arachis duranensis* | 25 | 6 | 1 | 8 | 40 | This study |
| *Arachis ipaënsis* | 27 | 8 | 1 | 8 | 44 | This study |
