# Supplementary material for: Genome-wide analysis of expansin superfamily in wild Arachis discloses a stress-responsive expansin-like B gene
Source: Plant Mol Biol. 2017 Feb 27;94(1):79–96. doi: 10.1007/s11103-017-0594-8 (PMC5437183; doi:10.1007/s11103-017-0594-8)
Supplement: Supplementary file 10 — Supplementary material 10 (DOCX 16 KB) [file 11103_2017_594_MOESM10_ESM.docx]

**Supplementary Table 4.** Type of duplication in *Arachis duranensis* and *Arachis ipaënsis* between the whole genome and the expansin superfamily.

|  | Duplication type | | |  |  |
| --- | --- | --- | --- | --- | --- |
|  | Dispersed | WGD*/Segmental duplication | Tandem | | Proximal |
| *Arachis duranensis* |  |  |  | |  |
| Whole genome | 68% | 17% | 8% | | 7% |
| Expansin superfamily | 24% | 55% | 11% | | 8% |
| *Arachis ipaënsis* |  |  |  | |  |
| Whole genome | 69% | 15% | 9% | | 8% |
| Expansin families | 27% | 50% | 18% | | 5% |

*WGD = Whole Genome Duplication
