# Supplementary material for: Genome-wide analysis of expansin superfamily in wild Arachis discloses a stress-responsive expansin-like B gene
Source: Plant Mol Biol. 2017 Feb 27;94(1):79–96. doi: 10.1007/s11103-017-0594-8 (PMC5437183; doi:10.1007/s11103-017-0594-8)
Supplement: Supplementary file 11 — Supplementary material 11 (DOCX 17 KB) [file 11103_2017_594_MOESM11_ESM.docx]

**Supplementary Table 5.** Genes involved in duplication events of the four expansin subfamilies in *Arachis duranensis, Arachis ipaënsis, Arabidopsis thaliana*, and *Glycine max*.

|  |  | **WGD*/Segmental duplication** | | | | | **Tandem duplication** | | | | |
| --- | --- | --- | --- | --- | --- | --- | --- | --- | --- | --- | --- |
|  | **EXPA** | **EXPB** | **EXLA** | **EXLB** |  | **EXPA** | | **EXPB** | **EXLA** | **EXLB** |  |
| *Arachis duranensis* | 65.2%  (15 of 25) | 33%  (2 of 6) | 0%  (0 of 1) | 50%  (4 of 8) |  | 4%  (1 of 25) | | 66%  (4 of 6) | 0%  (0 of 1) | 0%  (0 of 8) |  |
| *Arachis ipaënsis* | 59.25%  (16 of 27) | 25%  (2 of 8) | 0%  (0 of 1) | 50%  (4 of 8) |  | 7%  (2 of 27) | | 50%  (4 of 8) | 0%  (0 of 1) | 25%  (2 of 8) |  |
| *Arabidopsis thaliana* | 61.5%  (16 of 26) | 33.3%  (2 of 6) | 0%  (0 of 3) | 0%  (0 of 1%) |  | 23.1%  (6 of 26) | | 33.3%  (2 of 6) | 66.7%  (2 of 3) | 0%  (0 of 1) |  |
| *Glycine max* | 67.3%  (33 of 49) | 44.4%  (4 of 9) | 100%  (2 of 2) | 73.3%  (11 of 15) |  | 4.1%  (2 of 49) | | 22.2%  (2 of 9) | 0%  (0 of 2) | 46.7%  (7 of 15) |  |

*WGD = Whole Genome Duplication.
