# Supplementary material for: Genome-wide analysis of expansin superfamily in wild Arachis discloses a stress-responsive expansin-like B gene
Source: Plant Mol Biol. 2017 Feb 27;94(1):79–96. doi: 10.1007/s11103-017-0594-8 (PMC5437183; doi:10.1007/s11103-017-0594-8)
Supplement: Supplementary file 12 — Supplementary material 12 (DOCX 20 KB) [file 11103_2017_594_MOESM12_ESM.docx]

**Supplementary Table 6.** SNP frequency of 16 *AraEXLB8* genes from different *Arachis* genotypes.

| SNP | Fr (%) | Gene region | Position at chromosome of *A*. *duranensis* | Position at chromosome of *A*. *ipaënsis* | Frequent: Mutant base | BLOSUM62 | Genome type | | | |
| --- | --- | --- | --- | --- | --- | --- | --- | --- | --- | --- |
|  |  |  |  |  |  |  | A | B | K | AB |
| 1^c^ | 19 | E1 | A05:4844731 | B05:4927668 | G : A | -3 | 1 | 2 | - | - |
| 2^c^ | 6 | E1 | A05:4844753 | B05:4927690 | C : T | -3 | - | - | - | 1 |
| 3^b^ | 44 | E1 | A05:4844755 | B05:4927692 | T : G | 1 | 1 | - | - | 6 |
| 4^a^ | 6 | E1 | A05:4844766 | B05:4927703 | C : T | 5 | 1 | - | - | - |
| 5^a^ | 6 | E2 | A05:4845460 | B05:4928395 | C : G | 6 | - | 1 | - | - |
| 6^a^ | 13 | E3 | A05:4845631 | B05:4928567 | A : T/C | 6 | - | 1 | 1 | - |
| 7^b^ | 19 | E3 | A05:4845638 | B05:4928574 | G : T | 1 | 2 | 1 | - | - |
| 8^a^ | 6 | E3 | A05:4845691 | B05:4928627 | A : T | 7 | - | 1 | - | - |
| 9^a^ | 19 | E3 | A05:4845712 | B05:4928648 | C : G | 4 | 2 | 1 | - | - |
| 10^b^ | 6 | E3 | A05:4845716 | B05:4928652 | A : G | 1 | - | - | - | 1 |
| 11^c^ | 31 | E3 | A05:4845723 | B05:4928659 | A : C | -1 | 2 | 2 | 1 | - |
| 12^b^ | 25 | E3 | A05:4845732 | B05:4928668 | C : T | 0 | 1 | 3 | - | - |
| 13^c^ | 19 | E3 | A05:4845735 | B05:4928671 | A : T | -2 | 2 | 1 | - | - |
| 14^a^ | 6 | E3 | A05:4845745 | B05:4928681 | A : G | 5 | - | - | - | 1 |
| 15^a^ | 38 | E3 | A05:4845784 | B05:4928720 | C : T | 9 | - | - | - | 6 |
| 16^a^ | 6 | E3 | A05:4845811 | B05:4927747 | C : G | 4 | - | - | 1 | - |
| 17^a^ | 19 | E3 | A05:4845832 | B05:4928768 | C : T | 2 | 2 | 1 | - | - |
| 18^b^ | 50 | E4 | A05:4846088 | B05:4929024 | C : G | 1 | 2 | - | - | 6 |
| 19^a^ | 50 | E4 | A05:4846125 | B05:4929061 | C : T | 5 | 2 | - | - | 6 |

Sorts of SNP (Single Nucleotide Polymorphism): ^a^S:synonymous; ^b^NS: non-synonymous conservative; ^c^NC: non-synonymous non-conservative; E: exon; Fr: relative frequency (%).
