# Supplementary material for: Genome-wide analysis of expansin superfamily in wild Arachis discloses a stress-responsive expansin-like B gene
Source: Plant Mol Biol. 2017 Feb 27;94(1):79–96. doi: 10.1007/s11103-017-0594-8 (PMC5437183; doi:10.1007/s11103-017-0594-8)
Supplement: Supplementary file 13 — Supplementary material 13 (DOCX 23 KB) [file 11103_2017_594_MOESM13_ESM.docx]

**Supplementary Table 7.** Description of the single nucleotide polymorphism (SNP) on 16 *Arachis* genotypes. Green cells show the substitutions found along the 755 bp at *AraEXLB8* coding sequencing and their respective haplotypes.

| SNP number | 1 | 2 | 3 | 4 | 5 | 6 | 7 | 8 | 9 | 10 | 11 | 12 | 13 | 14 | 15 | 16 | 17 | 18 | 19 | Genome type | Haplotype | Life cycle | Resveratrol content^3^ |
| --- | --- | --- | --- | --- | --- | --- | --- | --- | --- | --- | --- | --- | --- | --- | --- | --- | --- | --- | --- | --- | --- | --- | --- |
| SNP position | 31 | 53 | 55 | 66 | 192 | 279 | 286 | 339 | 360 | 364 | 371 | 380 | 383 | 393 | 432 | 459 | 480 | 656 | 693 |  |  |  |  |
| *A. duranensis*^1^ | G | C | G | C | C | A | G | A | C | A | A | C | A | A | C | C | C | C | C | AA | H2 | A | G2 |
| *A. duranensis*^2^ | G | C | T | T | C | A | G | A | C | A | A | C | A | A | C | C | C | C | C | AA | H3 | A | G2 |
| *A. stenosperma* | G | C | T | C | C | A | T | A | G | A | A | C | T | A | C | C | T | G | T | AA | H6 | P | NA |
| *A. villosa* | G | C | T | C | C | A | T | A | G | A | A | C | T | A | C | C | T | G | T | AA |  | P | NA |
| *A. gregoryi* | G | C | T | C | C | A | T | A | G | A | A | C | T | A | C | C | T | G | T | BB |  | A | G3 |
| *A. cardenasii* | A | C | T | C | C | A | G | A | C | A | C | T | A | A | C | C | C | G | T | AA | H8 | P | G1 |
| *A. ipaënsis** | A | C | T | C | C | A | G | A | C | A | C | T | A | A | C | C | C | G | T | BB |  | A | G3 |
| *A. ipaënsis*** | A | C | T | C | C | A | G | A | C | A | C | T | A | A | C | C | C | G | T | BB |  | A | G3 |
| *A. magna* | G | C | T | C | G | C | G | T | C | A | C | T | A | A | C | C | C | G | T | BB | H7 | A | G2 |
| *A. batizocoi* | G | C | T | C | C | T | G | A | C | A | C | C | A | A | C | G | C | G | T | KK | H5 | A or B | G2 |
| *A. monticola* | G | C | G | C | C | A | G | A | C | A | A | C | A | A | T | C | C | C | C | AABB | H1 | A | G1 |
| Synthetic tetraploid | G | C | G | C | C | A | G | A | C | A | A | C | A | A | T | C | C | C | C | AABB |  | A | G1 |
| *A. hypogaea* - Caiapó | G | C | G | C | C | A | G | A | C | A | A | C | A | A | T | C | C | C | C | AABB |  | A | G1 |
| *A. hypogaea* - Runner | G | C | G | C | C | A | G | A | C | A | A | C | A | A | T | C | C | C | C | AABB |  | A | G1 |
| *A. hypogaea* - Tatu | G | C | G | C | C | A | G | A | C | A | A | C | A | A | T | C | C | C | C | AABB |  | A | G1 |
| *A. hypogea -* Tifrunner^+^ | G | T | G | C | C | A | G | A | C | G | A | C | A | G | T | C | C | C | C | AABB | H4 | A | G1 |
| Fs | 3 | 1 | 7 | 1 | 1 | 2 | 3 | 1 | 3 | 1 | 5 | 4 | 3 | 1 | 6 | 1 | 3 | 8 | 8 |  |  |  |  |
| Fr (%) | 19 | 6 | 44 | 6 | 6 | 13 | 19 | 6 | 19 | 6 | 31 | 25 | 19 | 6 | 38 | 6 | 19 | 50 | 50 |  |  |  |  |

SNP: Single Nucleotide Polymorphism; *A*.: *Arachis*; Fs: simple frequency; Fr: relative frequency (%). ^1^: *A. duranensis* accession K7988 (NCBI sequence number KX588115);^2^: *A. duranensis* accession V14167 (PeanutBase gene identifier number Aradu.MR104); *: *A. ipaënsis* accession KG30076 (NCBI sequence number KX588120); **: *A. ipaënsis* accession KG30076 (PeanutBase gene identifier number Araip.J65RE); +: (GO326087.1; (http://www.ncbi. nlm. nih.gov); H: haplotype ; A: annual plant, P: perennial plant; B: biennial plant; G: group; 3: Lopes et al. (2013). NA: not available.
