# Supplementary material for: Genome-wide analysis of expansin superfamily in wild Arachis discloses a stress-responsive expansin-like B gene
Source: Plant Mol Biol. 2017 Feb 27;94(1):79–96. doi: 10.1007/s11103-017-0594-8 (PMC5437183; doi:10.1007/s11103-017-0594-8)
Supplement: Supplementary file 14 — Supplementary material 14 (DOCX 17 KB) [file 11103_2017_594_MOESM14_ESM.docx]

**Supplementary Table 8.** Evaluation of transgenic (eGFP-positive) hairy roots on *Glycine max* composite plants.

| **Composite plant** | **Transgenic roots weight (g)** | **Transgenic roots (%)*** | **Number of gall in transgenic roots** | **Number of gall per weight (g) in transgenic roots** |
| --- | --- | --- | --- | --- |
| Empty-1 | 0.27 | 21 | 8 | 29.63 |
| Empty-2 | 0.07 | 5 | 1 | 14.29 |
| Empty-3 | 0.27 | 12 | 5 | 18.52 |
| Empty-4 | 1.27 | 39 | 9 | 7.09 |
| Empty-5 | 1.12 | 53 | 8 | 7.14 |
| Empty-6 | 3.05 | 76 | 45 | 14.75 |
| Empty-7 | 0.4 | 16 | 8 | 20.00 |
| Empty-8 | 0.17 | 10 | 7 | 41.18 |
| Empty-9 | 0.35 | 20 | 10 | 28.57 |
| Empty-10 | 0.54 | 18 | 15 | 27.78 |
| Empty-11 | 2.88 | 72 | 16 | 5.56 |
| Empty-12 | 1.72 | 52 | 18 | 10.47 |
| Empty-13 | 0.72 | 25 | 19 | 26.39 |
| Empty-15 | 1.42 | 87 | 20 | 14.08 |
| **Control Average** | **1.02** | **36.14** | **13** | **18.96** |
| AdEXLB8-1 | 0.41 | 36 | 0 | 0.00 |
| AdEXLB8-2 | 0.14 | 6 | 0 | 0.00 |
| AdEXLB8-3 | 1.13 | 51 | 1 | 0.88 |
| AdEXLB8-4 | 0.03 | 1 | 0 | 0.00 |
| AdEXLB8-5 | 0.28 | 10 | 1 | 3.57 |
| AdEXLB8-6 | 0.22 | 17 | 2 | 9.09 |
| AdEXLB8-7 | 0.58 | 35 | 2 | 3.45 |
| AdEXLB8-8 | 0.22 | 8 | 1 | 4.55 |
| AdEXLB8-9 | 0.23 | 8 | 2 | 8.70 |
| **AdEXLB8 Average** | **0.36** | **19.11** | **1** | **3.36** |

* Number of eGPF-positive roots per the total number of hairy roots in each composite plant.
