# Supplementary material for: Genome-wide analysis of expansin superfamily in wild Arachis discloses a stress-responsive expansin-like B gene
Source: Plant Mol Biol. 2017 Feb 27;94(1):79–96. doi: 10.1007/s11103-017-0594-8 (PMC5437183; doi:10.1007/s11103-017-0594-8)
Supplement: Supplementary file 15 — Supplementary material 15 (DOCX 16 KB) [file 11103_2017_594_MOESM15_ESM.docx]

**Supplementary Table 9.** *Arachis* genotypes used in this study.

| Genotype | Plant ID/Cultivar | Genome type |
| --- | --- | --- |
| Wild diploids | | |
| *A. batizocoi* Krapov. & W. C. Gregory | K9484 | K |
| *A. cardenasii* Krapov. & W. C. Gregory | GKP10017 | A |
| *A. duranensis* Krapov. & W. C. Gregory | K7988 | A |
| *A. gregoryi* C. E. Simpson et al | V6389 | B |
| *A. ipaënsis* Krapov. & W. C. Gregory | KG30076 | B |
| *A. magna* Krapov. et al. | KG30097 | B |
| *A. stenosperma* Krapov. & W. C. Gregory | V10309 | A |
| *A. villosa* Benth. | V12812 | A |
| Wild tetraploid | | |
| *A. monticola* Krapov. & Rigoni | V14165 | AB |
| Cultivated tetraploids | | |
| *A. hypogaea* subsp. *hypogaea* var. *hypogaea* | ‘IAC-Caiapó’ | AB |
| *A. hypogaea* subsp. *hypogaea* var. *hypogaea* | ‘Runner-IAC-866’ | AB |
| *A. hypogaea* subsp. *fastigiata* var. *fastigiata* | ‘Tatu’ | AB |
| Synthetic tetraploid | | |
| (*A. ipaënsis* KG30076 x *A. duranensis* V14167)^4×^ | AP Fávero 24* | AB |

*Herbarium CEN.
