# Supplementary material for: Genome-wide analysis of expansin superfamily in wild Arachis discloses a stress-responsive expansin-like B gene
Source: Plant Mol Biol. 2017 Feb 27;94(1):79–96. doi: 10.1007/s11103-017-0594-8 (PMC5437183; doi:10.1007/s11103-017-0594-8)
Supplement: Supplementary file 11 [file 11103_2017_594_MOESM6_ESM.docx]

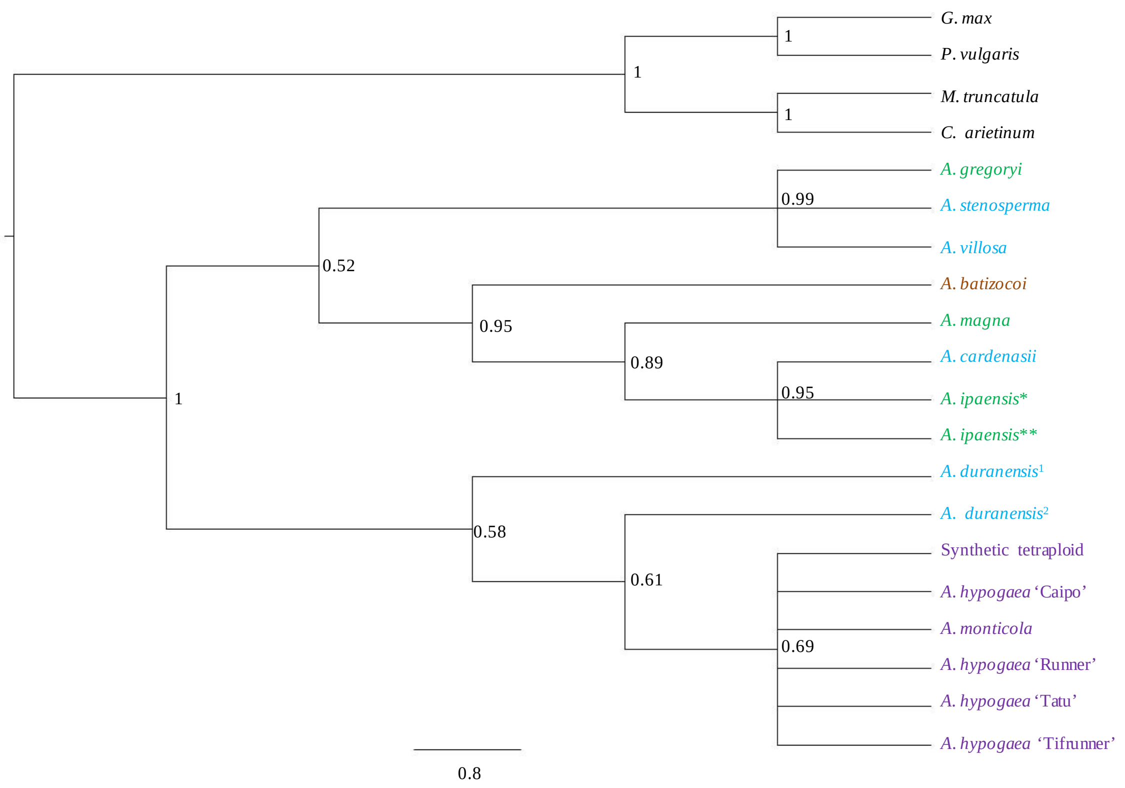
G. max
P. vulgaris
M. truncatula
C. arietinum
A. gregoryi
A. stenosperma
A. villosa
A. batizocoi
A. magna
A. cardenasii
A. ipaensis*
A. ipaensis**
A. duranensis1
A. duranensis2
Synthetic tetraploid
A. hypogaea 'Caipo'
A. monticola
A. hypogaea 'Runner'
A. hypogaea 'Tatu'
A. hypogaea 'Tifrunner'
1
1
1
0.99
0.52
0.95
0.89
0.95
1
0.58
0.61
0.69
0.8
